# Gender expectations, socioeconomic inequalities and definitions of career success: A qualitative study with university students

**DOI:** 10.1371/journal.pone.0281967

**Published:** 2023-02-24

**Authors:** Daniela P. Fernández, Michelle K. Ryan, Christopher T. Begeny

**Affiliations:** 1 Faculty of Health and Life Sciences, Psychology Department, University of Exeter, Exeter, United Kingdom; 2 Global Institute for Women’s Leadership, Australian National University, Canberra, Australia; 3 University of Groningen, Groningen, The Netherlands; University of Sharjah College of Health Sciences, UNITED ARAB EMIRATES

## Abstract

Higher Education (HE) is seen as a tool to create job opportunities and enhance individuals’ quality of life. Research demonstrates that students’ *expectations* of career success in HE are an important predictor of their motivation and academic attainment. However, there is a lack of clarity about how career success is *defined* and whether individuals perceive that their experiences (e.g., gender) may be associated with these definitions. In online written interviews with 36 university students in the United Kingdom, we examine how students define career success and how they perceive their identity (gender, socioeconomic status) experiences underpinning these definitions. We analysed three main definitional themes: (a) career success as personal development, (b) career success as individual mobility, and (c) lack of clarity about what career success is. Findings suggest that gender and socioeconomic experiences had an important role in students’ understanding of career success, especially for students from disadvantaged backgrounds. Indeed, in the intersection of gender and socioeconomic status, inequalities persist: female students anticipated difficulties in terms of work-life balance and gender stereotypes that constrained their career success definitions. Moreover, family experiences were important to understand students’ definitions of career success, particularly for disadvantaged socioeconomic groups. The current research sheds light on an important paradox in HE organisations: while students tend to define career success in relatively individualistic ways, such as individual mobility, financial success, or personal development, it was clear that their social identities (e.g., gender, socioeconomic status) and related experiences played an important role in creating definitions of career success. This further implies that when universities encourage a perception of career success as individual mobility, for example, having better job opportunities, or by espousing the belief that higher education and/or professional sectors are truly meritocratic–this will not always align with, and may create tension for, students from disadvantaged groups.

## Introduction

Globally, Higher Education (HE) enrolments have grown sharply in the past 30 years [1]. As one key aim of HE is to provide students with the skills needed to enter to the workforce and succeed in the labour market [2], HE is presented as a means to become more competitive in the job market, to avoid underemployment and unemployment, and to achieve social mobility [3]. In this way, HE is seen by many to facilitate success in life (e.g., greater employment opportunities, financial security and, in turn, a better quality of life), and universities are constantly evolving to be on top of the social, economic and job market demands [4]. However, this perception of HE does not always match reality. For example, in the UK, despite increasing enrolment in HE, unemployment rates for recently graduates are increasing, as is student debt [5]. Furthermore, precarious employment arranges for graduate professionals have increased [6], leading to job insecurity, lack of social protection for workers and negatively affecting employees’ mental health [7]. These trends undermine the notion that HE is a genuine or reliable vehicle for success.

For instance, some students–particularly women and individuals from lower socioeconomic backgrounds–experience less financial security or gains in employment [8]. Women with undergraduate degrees are paid less compared to their male counterparts, regardless of the university they attended or their academic outcomes [9]. The persistence of this gender pay gap is associated with the lack of female participation in disciplines perceived as more prestigious [10] and that have higher salaries [11]. Moreover, when disciplines became female dominated, the discipline experiences drop in its average earnings for men and women [12]. Women are also more likely than men to participate in part-time and temporary employment [13], challenging to have social protection rights or a stable income [14]. Furthermore, graduates from advantaged socioeconomic backgrounds occupy higher status careers that yield higher salaries [15], as they participate in more prestigious institutions [16].

The persistence of a belief in HE as a route to success despite evidence of unequal outcomes for disadvantaged groups leads us to pursue two questions that to our knowledge have not been deeply explored in the literature: (1) how career success is defined by HE students? and (2) how individuals’ social identities, particularly gender and subjective socioeconomic status (SSS), were associated by students with these definitions? Overall, research has focused on measuring expectations of success, but less attention has been paid to how individuals, particularly students, conceptualise what success is to them. For example, between 2015 and 2020, just one of 52 articles in Life and Sciences education focused on HE students’ own definitions of what success is [17].

Taking a social identity approach, we explore some potential ways in which individuals’ social identities (namely, gender and SSS) influence career success definitions -a key dimension of success to understand students’ choices in HE-, partly to allow us to understand the interaction between social contexts and individuals’ (multiple) social identities. We then report the findings of our study, grounded in a series of student interviews and an exploratory qualitative methodology, which allows us to recognise the potential nuances of the concept of career success, and how students’ definitions of career success are associated with their social identity-based experiences. Finally, we discuss the subthemes created from these interviews, and discuss implications for theory and practice.

### How to define career success?

A widespread approach to understanding the role of success in individuals’ experiences is through the concept of expectations of success. Expectations of success have been conceptualized as an individual’s general beliefs about the probability of success in a future task, activity or domain [18]; similarly see definitions in line with expectancy value theory; [19,20]. Within educational contexts, expectations of success influence a wide range of outcomes, including career choices [21,22], motivation to succeed [23], and career satisfaction [24].

However, research in terms of *how to define* success itself is less widespread [17]. Success has been defined as reaching one’s goals, accomplishing a task or, overall, when an individual’s outcome turns out well, desirable or favourable [25]. Although this definition can be applied to different situations, research about success definitions has traditionally focused on (a) career success, this is the achievements associated to individuals work experiences [26–28]; or (b) personal success linked to the individuals’ career [29].

Success can be measured in objective terms (e.g., a social or group metric) or subjective terms (e.g., an individual’s personal assessment; [30]). Although these objective and subjective terms can be interrelated [31], most research has concentrated on "objective" measures of success [32,33]. For instance, in HE research, students’ success has been defined as completing a degree [34], success in employment, earnings post-graduation, quality of life, and lifelong learning [35]. These wide-ranging definitions match with students’ own definitions of success, such as having leadership skills, financial success, and creating career networks [17,36]. However, school and university students also define career success from a subjective perspective, such as feeling happy, having others recognition, and being professional [36,37]. Hence, career success emerges as a key dimension of success research in HE [35].

However, broad definitions of success might lead to the exclusion of the role of social context in how individuals define success, and particularly career success. Contextual factors such as group memberships, socialization, and stereotypes may have been overlooked in success expectations research (see [38]), as well as career success research. For example, married Korean women in non-managerial positions were more likely to understand success as job security and work-like balance, compared to unmarried Korean women. This difference between married and unmarried women can be associated with gender expectations regarding women’s role and stereotypes shared by employers that married women are less productive workers compared to men [32]. Moreover, previous research looking at how individuals understand career success has shown that men in male-dominated professions defined success in terms of material/financial outcomes, implicitly including the stereotypes of men as “provider” and “breadwinner” [21,22], and as risk taking and intelligent [39] in their definitions. Therefore, women that conceptualise career success in a male-stereotypical way (e.g. economic success, financial mobility) may be seen as violating gender stereotypes prescriptions [40] and, in turn, change their career success definitions to a more female-stereotypic conceptualization, associated with having a family and positive relationships [41], or having social support and feeling to belong [26], which are less valued in society compared to success in the workplace [42].

Additionally, women in male-dominated professions defined personal and professional success in terms of internal criteria, including personal recognition and work-life balance [43]. However, women in the same job position but from different cultural contexts might conceptualise success differently. For example, women in executive positions in Brazil defined success as “academic mobility”, that is, having a degree that allows them to reach higher work positions [44]. Therefore, to navigate gender expectations and inequalities at work, it is likely that for women the relationship between subjective and objective measures of success is perceived as a continuum, rather than as a rigid dichotomy [45]. Moreover, to maintain definitions of career success in terms of financial success or mobility, which have become hegemonic in HE organisations [17] can also promote that individuals that already have wealth and prestige see success as more attainable, compared to individuals from disadvantaged socioeconomic backgrounds that do not have the same economic and cultural resources to achieve success [46].

For instance, research looking at career success definitions/conceptualisations has focused on professional/high ranking individuals, such as executives, who tend to define success linked to their career in terms of happiness and personal development and place less importance on financial circumstances, in contrast with those in less socioeconomically privileged groups (see [43]). For less socioeconomically privileged groups, financial circumstances might be more important as they have not been covered/taken for granted during their life. Financial circumstances might be perceived as a first step to reach happiness [26] and, therefore, are prioritised by socioeconomically disadvantaged groups and can be a way to reach happiness (for an analysis of the relationship between happiness and financial circumstance see [47,48]).

In educational contexts, economic status has also been associated with differences in the way that individuals define success. Curiously enough, as research about career success definitions has focused mainly on gender differences, research about students’ definitions of success (either general, career or academic success) has mainly focused on economic status differences. Research from the 1960s showed that students defined educational and career success differently according to their social class, with middle and upper class participants defining success as status, prestige, and personal worthiness, while students with unskilled manual labour fathers framed success as being wealthy [49]. In fact, more recent research in educational contexts, has shown that students from privileged socioeconomic groups define success including personal and career dimensions, and as having the financial resources to be able to enjoy travelling, holidays, a good education for their children, and buying a house: a salary just to cover basic expenses would not be enough to be considered successful [28].

Additionally, parents without a university degree had higher expectations on students’ achievements and outcomes of having a college degree, associated with financial success and social status [50]. However, research has also shown that parent socioeconomic background might cause guilt in students when their ideas about success are different to their parents’ expected trajectories for them, or leading to insecurities about their future and taking risks in their career choices [50].

Success is a concept built in individuals’ experiences [31], which are subjective and multiple. Hence, career success definitions are associated with group-based experiences, yet these definitions have different nuances for individuals depending on the group to which they belong, and the intersectional nature of these identities. In this way, definitions of career success are neither static nor fixed. For example, even though women’s definitions of career success have been shown to be associated with gender norms and stereotypes about women, for Arabic women, career success is seen as an act of rebellion against gender norms and stereotypes [51]. Historically gender and ethnicity have been associated with creating different ideas about what career success is for women, such as the idea of white women in professional settings and women of colour as workers in service sectors [52].

Hence, intersectional experiences of disadvantages will promote different approaches toward career success and how to define it. For example, to succeed, working class Chinese immigrants in the US need to rely on their co-ethnic networks, which can be limited [53]. These barriers to pursuing their goals shape how individuals define career success: women from minoritised ethnic groups are aware of their restricted access to informal networks that help other individuals’ career progression—such as women from majoritised groups [54]. Therefore, definitions of success, and particularly career success, are not homogeneous concepts, but rather, built on diverse and intersectional identity-experiences.

### A social identity approach to understand intersectional groups’ definitions of success

As we mentioned above, gender and socioeconomic background have shown to influence individuals’ *levels* of career success, and also how career success is *defined* by individuals. However, individuals’ identities do not occur in a vacuum and are shaped by their relationships with others and their social context [55]. Indeed, one potential key determinant of individuals’ definitions of career success are their social identities (see [56]).

Given the importance of identity, the social identity approach [57] provides a useful theoretical framework from which to examine career success. The social identity approach demonstrates how one’s social identity is constructed by a sense of membership within a social group. People tend to classify themselves, and others, into social categories and groups, and this classification process impacts on individuals’ behaviour.

The social identity approach also outlines a series of identity strategies that are relevant to notions of career success. An individual’s position in the social structure is not static. Rather, individuals can try to pursue a better position (status) through social creativity, social competition and individual mobility [57]. Which strategy an individual chooses depends on perceived characteristics of the social context, such as the permeability of group boundaries [58]. Perceived permeability of a group’s boundaries would signal to low status group members that individual mobility is possible, decreasing the identification with their group. Indeed, the perception of permeability is likely to promote individuals’ aspirations for individual mobility [59].

Social identities are subjective and depend on individuals’ subjective sense of belonging to different groups. On this basis, we (a) understand gender as a social identity that results from a self-categorisation process, rather than based on or correlated with biological sex [60]; and (b) frame socioeconomic status as subjective social status (SSS), highlighting the fact that individuals’ sense of their socioeconomic status is situated in particular contexts, and their identities are based on their subjective perceptions relative to others [61]. Hence, we understand social identities as subjective and therefore focus on individuals’ subjective sense of belonging to different groups.

Moreover, individuals not only categorise themselves in certain groups, but also evaluate their groups compared to others through social comparison [62]. For example, if women or low SSS students perceive a lower fit between themselves and the model of career success presented as possible in their context (e.g. family or university), this may influence their individual behaviours and future choices [56]. For instance, the definitions of success from middle class men in prestigious programmes (e.g. law, medicine and engineering physics) match with what universities have offered as “successful” in these programmes -male, effortless, relaxed attitude and superior in intelligence [33].

Thus, as students that fit in this model can feel confident that success in those terms is attainable for them, they might focus on other conceptualisations of career success as important, such as personal growth or personal recognition. In other words, individuals from less privileged groups (such as women and low SSS students) might conceptualise career success as reaching financial and individual mobility, as they may tend to define success in ways that emphasise what generally have less of. On the contrary, individuals from privileged groups (such as men and high SSS students) may tend to conceptualise career success including more internal aspects of success, such as personal development and happiness, as they may already have a sense of career success in financial and individual mobility terms or, at least, it is perceived as more attainable. Similarly, low SSS students have been shown to choose “less ambitious” careers [63], but this may be explained, at least in part, by students applying to universities where they can see success as possible and where they feel that their conceptualisations about success are achievable.

Hence, to understand students’ definitions of career success, it is important to consider the different social identity groups in which students participate, as they will provide a sense of -or lack of- potential and realistic success options, and create particular career success definitions. We can expect that students’ definitions of career success may vary as a function of their social identity-based experiences. For example, when individuals’ career success level differences have been researched, gender and socioeconomic status are included as key variables to understand these differences. However, most of this research (a) has been conducted exploring gender differences within professional participants (see [22,27]) or socioeconomic differences in within students conceptualisations of success (see [28]); (b) has focused on social identities as demographic variables, and on quantifying differences between social identities; and (c) has explored the roles of intersectional gender and ethnicity (e.g., [45,64], and social class and ethnicity experiences [52] to understand how students define career success. Currently, little is known about how students themselves (a) define what is career success; and (b) associate their gender and SSS identity experiences with how they define career success. Hence, our study aims to cover this vacancy, analysing students’ definitions of career success, and the role of their gender and SSS experiences in these definitions.

Given the evidence for the roles of both gender and SSS as social identities that influence conceptualizations about career success, it is important to understand how these two social identities may intersect. Thus, while we know that gender and SSS shape individuals’ career choices, less is known about how individuals experience the role of the *intersection* of gender and SSS in their *definitions* of career success. Most of the research about career success definitions has been conducted with women in professional or male-dominated positions -which are perceived as more prestigious-, leaving the question of how women outside this group (e.g., from socioeconomically disadvantaged backgrounds) and as students might conceptualise career success.

Both social identity [56] and intersectionality [65] perspectives recognise that social groups are hierarchically organised, and that this is important for understanding how individuals are perceived and treated within social structures. The social identity approach has traditionally focused on the hierarchical position of individuals based on a single social identity (e.g., based on an individual’s gender) or multiple identities where one becomes salient in particular contexts [66], paying less attention to the idea that intersectional identities (e.g., an individuals’ gender and their social class) come together in a way that generates a more specific position within that social hierarchy [67]. At the same time, intersectionality has offered this more nuanced lens for recognising individuals’ positions within social hierarchies, but has not offered the same level of detail in how individuals might experience that social hierarchy.

Specifically, by considering social identity principles through an intersectional lens–both conceptually and analytically–we can more thoroughly consider how multiple social identities overlap in terms of their positions and experiences of social disadvantage or privilege (e.g., being a female student from a low SSS background vs. a female student from a high SSS background). This integration of social identity and intersectionality perspectives lends itself to generating a more nuanced understanding of how students approach career success. Eventually, this is why, when aiming to comprehend individuals’ definitions of career success, it is critical to adjoin insights from both social identity and intersectionality perspectives.

Moreover, to share different group memberships might promote nuances in how students define career success. For example, a female student with a high SSS might perceive career success as financial and happiness, yet one element can be referred as more important, depending on which identity is more salient in particular contexts, creating particular perspectives about career success. Therefore, the term intersectional identity will refer to social identities that are constructed by a membership of social groups and categories, and that result in political, social and economic consequences for the individual [68].

### The current research

Success has been a key research topic within psychology in the past decades. Most of the research has focused on career success, as it has been demonstrated that success expectations are associated with individuals’ motivation and ambition. This is particularly important in educational settings, where individuals enter to pursue a degree that allows them to maintain or improve their life conditions. However, less research has focused on how individuals, especially students, define career success, and how they might associate their social identity (gender and SSS) based experiences with these definitions. Social identity-based experiences may provide nuances and heterogeneity in understanding how career success is conceptualised, which can contribute to understand students’ motivation, future career choices and job applications, as career success conceptualizations will be related to applicants’ choices to particular jobs and organisations. Indeed, compared to research looking at career success outcomes and different operationalisations, research focused on how individuals define/conceptualise career success is minimal. Hence, it is important to acknowledge the diversity of career success definitions and understand how the context where students create these definitions might be related to their conceptualizations about career success.

In the current research, we have two core aims: (a) to examine HE students’ definitions of career success, and (b) to analyse if and how students associate their gender and SSS social identity-based experiences with their definitions of career success. Following this, we interviewed UK female and male university students with different SSS, to provide an understanding of students from different gender and socioeconomic backgrounds conceptualise career success. Hence, rather than test explicit hypotheses, our study aims to generate insights about an existing gap in the current literature. In the interviews, students referred to different approaches to understanding career success, from tangible outcomes (e.g., financial) to intangible outcomes linked to their career (e.g., personal development, happiness), and -to different extent- their gender, SSS or intersectional experiences played an important role in the way they understood what career success is.

## Methods

We used real-time, semi-structured online written interviews via a document sharing website (Microsoft Outlook), following Opara and colleagues [69]. Compared to face-to-face interviews, online written interviews have the advantage of following a communication format widely used by HE students, which they might find more familiar, as text messaging is a key method of communication among HE students [70]. Typing answers can also provide the participant with a sense of anonymity and confidentiality, as there is physical distance and participant and interviewer cannot see each other, decreasing the bias between both [69]. Moreover, this method also enabled us to access students from diverse locations within the UK, as participants could access the interview from any place with access to internet in any city within the UK.

Interviews were semi-structured and formed part of a more extensive study about students’ university experiences. We followed the methods in Fernández and colleagues [71]. This study was approved by the first author institution. Participant consent was written and obtained electronically.

### Participants

We interviewed 36 undergraduate students enrolled in UK universities. The sample included 19 women and 17 men. Gender was obtained from students’ self-report. Participants’ mean age was 21.99 (*SD* = 3.39), and, on average, they were enrolled at 2.51 years of study (*SD* = 0.731). The initial call for participants focused on students from 2^nd^ year and above, to interview students with more experience with university life. However, to recruit more participants, we opened the call for students from all years. We gathered students’ SSS into 3 groups considering the median (5.5.), and clustering groups around the highest and lowest levels around the median: 12 students in the lower SSS group (values of 1–4), 10 students in the mean group (values of 5–6), and 14 students in the higher SSS group (values of 7–10). Participant attended different UK universities and were enrolled in a variety of disciplines (see Table 1). Although we asked students for their university and discipline, these variables were not included in the analysis, as we focused on the role of their gender and SSS experiences (see Limitations and future research section).

**Table 1:** Demographic data of participants.

Pseudonymous	Age	Gender	Year	Subjective social status
P1	21	Woman	3	Low
P2	21	Woman	3	Low
P3	21	Man	3	High
P4	20	Woman	2	Mean
P5	19	Woman	2	Low
P6	32	Woman	2	Mean
P7	21	Woman	2	Mean
P8	22	Man	4	Mean
P9	19	Woman	2	Low
P10	22	Woman	4	Mean
P11	20	Woman	2	High
P12	20	Woman	2	Low
P13	21	Man	3	High
P14	22	Man	3	Low
P15	28	Woman	4	High
P16	20	Woman	3	Low
P17	19	Woman	3	High
P18	27	Woman	2	Mean
P19	20	Woman	2	High
P20	31	Man	2	Low
P21	21	Man	2	High
P22	19	Man	2	Mean
P23	19	Man	2	High
P24	28	Man	3	Low
P25	20	Woman	3	High
P26	20	Woman	2	High
P27	21	Man	3	High
P28	23	Man	3	High
P29	19	Man	2	Low
P30	20	Man	3	High
P31	26	Man	3	Mean
P32	21	Man	3	Low
P33	21	Man	2	Low
P34	22	Woman	1	Mean
P35	19	Man	1	High
P36	26	Woman	3	Mean

In the following sections, we will describe: (a) participants’ recruitment process; (b) how interviews were conducted; and (c) analytical procedures.

### Process

We shared a brief demographic screening questionnaire through the online participant recruitment site Prolific, Facebook student groups, and university contacts from Widening Participation programmes. We purposely used multiple recruitment platforms to capture a greater scope of students. The pre-screening questionnaire was designed to establish eligibility and collect key demographic data. It included self-reported questions about students’: (a) gender; (b) field of study; (c) university; (d) parental education status; (e) household income; (f) year of study; and (g) whether they would like to be contacted for a future online written interview. We also included one question to collect students’ SSS using the MacArthur Social Class Ladder (adapted from [72]), where participants identified their place relative to people in the UK. After confirming their interest in participating in the interview, students received an interview invitation (depending on how participants were recruited, via Prolific or email) and the participation information sheet. As the interviews were conducted online, we could share a wide range of option schedules and also include an option for students to propose their availability if it was not included on the list. Afterwards, each participant received a unique link to access the online document. The link was shared via private message or email one day before the arranged interview time, with a reminder message about the interview.

Following Braun and Clarke [73] on the use of saturation in reflexive thematic analysis, the sample size was not determined a priori. Hence, participants were selected through quota theoretical sampling [74]. We started by interviewing between 10 and 12 participants, aiming to have an equal number of women and men. After this, we contacted students while maintaining an equal number of participants for each category. We stopped recruiting more participants when (a) the topics mentioned in the interviews addressing the research question started to repeat and overlap, and (b) we reached a roughly equal number of participants in each gender and SSS group (See S1 Table).

The online written interviews were semi-structured and consisted of a thematic script that included: (a) how they define success; (b) how they define being successful in their career; (c) their expectations after graduation; and (d) whether their gender or social class experiences have affected their success expectations (see S2 Table for the Interview script). We include probing questions to clarify and elicit more detailed responses when needed, such as (a) Can you give some examples? (b) Why do you have that perception? (c) Why do you think that? and (d) What do you mean by (…)?

The first author typed the questions live into the document and participants typed their responses in reply. We were able to ask follow-up questions for a better understanding of the participants’ initial responses, replying to their answers on the same shared document during the interview (see [69]). Each interview lasted approximately 2 hours and could be conducted in one or two separate sessions (in the latter case, within the same week). Within these 2 hours, as the interviews were part of an extensive research about students experiences in HE, other topics were discussed but not included in this study. As the interviews were conducted via an online document, the answers to the questions were written by the participants.

Students were debriefed via email and received payment for their participation in line with the national minimum wage. We then anonymised names, institutions, cities, and third parties within the transcription.

### Analytical procedure

We followed a qualitative and interpretive approach to analyse participants’ definitions of career success, following a reflexive thematic analysis approach [73]. Using an “analytical sensibility” [73], we approach the data from a social identity perspective [57], acknowledging how the social context shapes students’ multiple and intersecting identities [65,68].

We analysed the data following the process proposed by Trainor and Bundon [75]. First, after each interview, the first author read the transcript in detail to identify relevant data and discussed it verbally with the second author. Next, after all interviews were conducted the first author read through each participant’s transcripts again. Then, the first author created reflexive notes about potential meanings and patterns, using NVivo (version 12, 2018). The first author began analysing each interview at a time, considering what participants had explicitly said. The first author then re-read each interview and review the list of initial codes (see S3 Table) and create the final list of codes. The new list of codes collapsed previous codes as they were redundant (e.g., “success as helping people” and “success as helping normal people”), and excluded codes that were considered not related to the research question (e.g. “taking time off”).

Following this, the first author grouped the codes in subthemes and themes following an inductive analysis, considering the data following the research questions: (a) in which ways students define career success? and (b) how students associated their gender and socioeconomic based experiences with these career success definitions? The first author developed themes and subthemes according to the research questions, constructing relationships among the codes within the data set. The codes, subthemes and themes were constructed as mutually exclusive, that is, a code could not be part of two different subthemes (the same for subthemes and themes).

Afterwards, the list of themes, subthemes, and codes was shared with the second author as a hierarchically organised table for comments. We followed this approach considering Braun and Clarke’s [73] comments about the coding process as collaborative. After verbally discussing the table, the first author reviewed the transcriptions, codes, subthemes, and themes again, interpreting the material following an intersectional social identity approach (deductive analysis) and making changes in the names of the subthemes to make them more representative of the material (for the coding process development, see S4 Table). The analysis stopped when we could not identify alternative patterns with the codes and subthemes created to address our research questions. Nevertheless, following reviewers’ comments, we reviewed the subthemes and themes and now present the final version. All the coding process was made using the software NVivo so we could automate the data processing.

## Findings

Our analyses produced three main themes reflecting how students defined career success: (a) career success as *personal development*, (b) career success as *individual mobility*, and (c) *lack of clarity* about what career success is. Students’ definitions of career success followed an individualistic approach and transitioned between internal (e.g., happiness) and external (e.g., finances) references, and these differences were explained particularly by personal experiences related to their SSS and gender norms. For example, students who had experienced socioeconomic barriers defined career success as improving their status, and this way, boosting a positive identity through individual mobility. However, within this group, individual mobility appears as more challenging for women, as they recognised the challenges of work-life balance associated to their gender. Furthermore, a minority of students also described an uncertainty about what career success means or how to pursue success considering contextual circumstances, such as the Covid-19 pandemic. We will consider each of the themes in turn. We have also provided a summary of each theme including their respective subthemes and codes, and example quotes (see Tables 2, 3 and 4).

**Table 2:** “Career success as personal development”: Subthemes, codes and quotes.

Quote	Codes	Subtheme	Theme
*‘I would view success as the ability to improve the lives of other people*, *compared to the vision some people have of a large bank balance equating to success*. *If I can change someone’s life for the better then that would be the ultimate level of success’*. *(P20*, *Man*, *low SSS)*	Improve others people lives	Career success as meaningful experiences: The role of previous socioeconomic experiences	**Career success as personal development**
*‘Success to me means that I have found something which is meaningful*. *I would consider myself successful if I were pursuing something meaningful and which was generating a decent livelihood for me*. *If*, *for example*, *I had a particular area of law that I felt was profoundly worthwhile studying and becoming an academic for*, *that is success (at all stages of the career)*. *Success is not pecuniary in nature*, *but this can come into it*. *(Man*, *low SSS)*	Meaningful experiences		
*‘I think depending on the work itself*, *I still expect to find friendly colleagues*, *as I am a person who values good relationships around me’*. *(Woman*, *mean SSS)*	Workplace relations		
*Success for me is being able to say I achieved something and that the hard work I am most of the time putting in has given me better understanding and knowledge (P15)*	Success and personal grow		
*‘Being successful in my career would be finding a job that I really enjoy and am happy doing whilst working hard to make a difference’ (Woman*, *high SSS)*	Being happy	Career success as happiness	
*‘I believe a good attitude is very important*. *You have to be positive*, *optimistic and respect*. *Discipline is a major factor because discipline helps you keep your eyes on the prize and focus on tasks*. *Determination helps you not to throw in the towel when things get difficult*. *Last focus helps you to be less distracted’ (Woman*, *high SSS)*.	Positive mindset		
*‘In my career being successful right now is finding a job in the future that I enjoy doing regardless of the payment that I am earning’*. *(Man*, *mean SSS)*.	Work as a source of happiness		

**Table 3:** “Career success as individual mobility”: Subthemes, codes and quotes.

Quote	Codes	Subthemes	Theme
*‘Honestly speaking*, *I have yet to decide if finance is for me completely*, *but I a very fond of mathematics*, *numbers and earning money haha*, *so those 3 things combined*, *as well as how huge the industry is*, *and how it combines all aspects of the world*, *like any news of the world can have an impact on the markets*, *so you have to be on top of everything*. *How is being fond of earning money*? *The finance industry compensates quite well’*. *(P3)*	Career choices	HE as tool for career success	**Career success as individual mobility**
*‘My University has helped me in realising my potential and opened up a whole whole world of opportunities for me*. *Making me realise that I can achieve more that I had my mind set to’*. *(P36)*	University shapes success		
*‘I won’t prioritise money greatly over my social life/mental health but I feel like it is a big thing because everybody wants to earn money*, *right*. *Like we don’t go to uni*, *which is optional*, *and spend 3 years doing countless exams and have so much student debt to not be earning money once we get jobs (P9)*.	Education as an investment		
*‘So being successful can be having a stable job that you do but don’t necessarily feel fulfilled by*, *but that enables you to pursue your hobbies for example’ (P7)*.	Having a stable job	“I did not grow up rich”: Career success as financial security	
*I think its partly due to the expectations my parents put on me growing up*, *I grew up thinking I would always go to university so when it came to applying I didn’t care about where I went and what I learnt as long as I could say “im a uni student” the actual uni didn’t really come into my decision*. *(P29)*	Family expectations		
*‘I think*, *in regards to my career*, *I define success in various stages*. *The first being getting a job*, *in general*, *in a field of my interest and doing a good job*. *Then*, *after that*, *success would be continuously working my way “up” until*, *hopefully one day*, *I’d achieve my own ultimate goal of being a lead researcher in a study’*. *(P12)*	‘Working my way up’		
*‘I guess I am also thinking of the future*, *because my course is four years long*, *what if me and my partner decided to start a family*, *would I be able to continue with my studies*. *If I did continue*, *I feel there would be a lot more stress and worry (only if this situation occurred)’ (P18)*	Success as work life balance	A gendered definition of career success: work-life balance	
*‘My dad initially wanted me to study business but I did not have an interest so at first it was a bit difficult to understand what I am studying but once he knew I was happy studying it he was happy for me too’*. *(P16)*	Career choices and family expectations		
*Being from a very conservative family means*, *as a woman*, *I should be married and giving birth to children and not studying*, *so I sadly do not have the financial support from my family here*. *(P12)*	Gender roles and family lack of support		

**Table 4:** “Lack of clarity about what career success is”: Subthemes, codes and quotes.

Quote	Codes	Subthemes	Theme
*‘I have open mindset as I understand the world is in a strange place right now’*. *(P7)*	Social context as a source of success instability	Career success and perceptions of unemployment: Social and contextual constraints	**Lack of clarity about what career success is**
*‘Im not sure*, *it seems like its getting harder and harder for young people out of university to go into the jobs they want and you hear a lot about people with degrees working random jobs that don’t pay very well’ (P5)*	Concerns about job market		
*“Hard work is subjective*, *results are objective” I still haven’t really understood success to be honest*. *(P35)*	Don’t understand success	Unclear definition of career success	
*‘I think the future will decide that for me*, *once I find the job that I enjoy*, *I will look back at the journey that brought me to that situation’ (P8)*	No expectations of success		

### Career success as personal development

Students considered career success to be the achievement of intangible rewards with intrinsic value beyond economic incomes [23], with a focus on internal comparative references. More specifically, a group of students defined career success in terms of worthwhile experiences, happiness, and personal growth, with a lack of intention to identify themselves with different higher status social identity groups. However, the motives behind this definition were different according to their SSS experiences. Particularly those who had faced challenges due to their background, especially in socioeconomic terms, described helping others as a goal specifically because they had not received the same support previously, which is an example of social creativity strategies [57]. Thus, having fewer opportunities in the past motivates them to help others, compared to people that had better opportunities and may be less aware of the importance of these variables (in this case, high SSS students). Therefore, previous experiences of disadvantages, especially on a socioeconomic dimension, were mentioned as an explanation of particular career success definitions.

### Career success as meaningful experiences: The role of previous socioeconomic experiences

A group of students defined career success as having a job that becomes a source of worthwhile experiences, especially in term of the relations developed with others. This was seen particularly in terms of helping people as part of their job. For example: *“I don’t want to work with athletes but rather normal population and use exercise and nutrition to make life better for them”* (P3; man; high SSS). For these students, helping others provides a sense of positive identity and boosts their motivation to have a degree, while a feeling of being successful at work implies a certain status relative to those who are helped, as at the same time this altruism provides a reward to the helper.

Furthermore, for these students, perceiving career success as being in a position to help others was related to their previous lack of support in their personal experiences. More specifically, being able to help was spoken about in relation to themselves not being helped previously. To some extent, this can be understood as a form of social creativity [57], as students from low status groups (e.g. women) re-interpreted their relationship with high status groups reading their difficulties as a positive identity resource (being able to help others). For example: *“I believe success is taking what you have learned from uni [sic] and sharing it with people you meet in the future*, *e*.*g*. *if I become a teacher I will share my experiences and advice as in my high school I did not have a lot of support from my teachers”* (P26; woman; high SSS). Thus, it can be argued that to focus on helping others as a conceptualisation of being successful is promoted by students’ perception of a stable social context and, thus, to reach a positive identity, they will need to change the comparison parameter with other groups, rather than “move” to higher status groups (see [76]).

### Success as happiness

Other students defined career success as being happy, either as a goal or as a resource to reach success: *“Being successful in my career would be finding a job that I really enjoy and am happy doing whilst working hard to make a difference”* (P11; woman; high SSS). Following this, students also reported that to achieve career success, one must be happy before starting a job, as this happiness will provide a better attitude at work, more happiness and, finally, a sense of success: *“You have to be positive*, *optimistic and respect”* (P17; woman; high SSS). Seeing happiness as a means to reach career success may be interpreted as a part of a meritocratic belief system, whereby individual effort and attitude are key for success.

Hence, career success was described as a way of reaching personal empowerment: *“If you constantly assume you aren’t good enough and can’t do something*, *then you will lack motivation*, *lack self-belief and lack confidence to really push yourself to the limit to see what you can achieve”* (P17; woman; high SSS).

### Career success as individual mobility

As students defined career success in light of their background, particularly that based on familial ties and socioeconomic status, career success was defined in terms of individual mobility, that is, to move from their group to join a higher-status, more valued group which, in turn, boosts their positive identity [57]. Indeed, some students defined HE as an investment towards future job opportunities that will secure them a better life. Particularly, when students have faced socioeconomic barriers, career success was associated with employability and economic stability, especially following their family experiences. However, this association between financial outcomes and success also led to female students that perceived themselves with a lower socioeconomic status to acknowledge how challenging this career success outcome can be for them, especially in terms of gendered norms and work life balance.

### HE as a tool for career success

Students considered HE as an instrument to reach, or maintain, higher social status and as a strategy to navigate a competitive job market. This supports previous research that demonstrates that students from disadvantaged background see HE as a tool for social mobility and career success [51]. HE is considered evidence of what social structure offers in terms of permeability of group boundaries, as universities themselves present to students as being committed to improving students’ social mobility and enhancing their lives [77]. Therefore, their academic choices are evaluated by considering future financial prospects. As career success was defined in financial terms, for men in particular, their career choices were made to provide a pathway to future financial success and employability, even if this meant not following careers that they may enjoy more: *“My real passion was art*, *but I went with better career prospects with the computer science degree”* (P24; man; low SSS).

However, these choices were not equal for all, and were particularly constrained for anticipated experiences of discrimination due to their gender and socioeconomic status: *“(…) I chose this university because it was good but not like top 5 or 10 if you know what I mean*. *And it’s been a good decision because I personally havent [sic] experienced any discrimination based on my gender/class”* (P9; woman; low SSS). Indeed, students who did not mention discrimination (either anticipated or current) related that university was a tool/mean to reach career success, describing a more strategic approach in their career choices compared to students from disadvantaged backgrounds who chose less prestigious institutions because of their fears of facing discrimination. Hence, these students considered the status of their university as potential disadvantage for them, suggesting that they may sacrifice their status to avoid discrimination, constraining their career choices. Strikingly, the universities in which they think they will fit may not provide them with the same resources or access to the workforce -and, therefore, might constrain students’ definitions of career success- that higher status universities may offer (see [63]). Thus, previous success expectations, as well as the anticipated identity cost of these expectations (e.g., feeling that they might not belong) will shape students’ career/university choices which, in turn, will shape their career success definitions.

The role of university as a tool for reaching career success was extended beyond undergraduate studies. Students discussed their motivation to enrol on postgraduate courses with the purpose of having better job opportunities and differentiating themselves from other students with better opportunities or traits to get a job: *“It’s hard to get a job in psychology at the moment without a masters and so success at university helps me get my degree*, *which helps me get onto a master’s course*, *which then helps me get a job down the line”* (P10; woman; mean SSS). However, these opportunities were also constrained by socioeconomic status and economic resources, leading to a sense that economic resources are needed to accomplish their career success goals: *“My peers don’t have these issues and I notice they just apply for whatever [Masters] catches their eye*, *I find that this also makes me feel out of place when they ask about where I’m applying to”* (P2; woman; low SSS).

### “I did not grow up rich”: Career success as financial security

Low SSS students defined career success in terms of making it to the top of their future organisation. They shared a career-orientated idea of career success in which financial success is important, showing tensions between admitting to have more “materialistic” goals and more optimistic, ungreedy goals (for a review of the tensions between economy and wellbeing, see [47]): *“I measure success on a big mixture of happiness and money with a big weight on happiness–but money certainly will ease those worries won’t it”* (P1; woman; low SSS).

Social identities-based experiences constrained students’ choices to reach success, as well as their definitions about career success. Indeed, career success definitions were shaped by contextual factors, and particularly by students’ family background. Hence, family financial circumstances promoted a definition of career success that had its basis in earning a good salary and, therefore, not having to face the same economic struggles as their family: *“(…) that means they [family] have had to work manual or low paid jobs for most of their lives and I think looking at them and seeing this*, *Ive [sic] always really strived to work hard and study hard to build as good of a life as possible”* (P2; woman; low SSS).

Furthermore, students reported that overcoming their economic position would make their family proud: *“Wanting to better my career options and to make family proud of me”* (P18; woman; mean SSS). Likewise, career success was strongly defined as improving not only their financial circumstances, but also that of their family: *“I feel like my ultimate goal in life is to be able to repay my mum for everything she’s done for me*, *I want to be able to buy a house instead of a flat*, *and for all those goals you need money”* (P9; woman; low SSS).

Family background also shaped students’ definition of career success as relative compared to other students’ family backgrounds. As students compared themselves with others at university, their SSS identity shaped the strategy approached to improve their social identity–particularly for those students who identify as “being a first-generation student”:

*“My housemate is from a wealthy family with a long line of academics and doctors before him*, *but my father is a welder and my mother is a cleaner*. *If I become a lawyer I will feel really successful*, *but if my housemate becomes a doctor he will feel he has done the bare minimum”* (P14; man; low SSS).

As the interviews illustrated, family circumstances are important in terms of career choices that allow students to reach career success, particularly financial success, as well as the parameters with which are assessing success.

### A gendered definition of career success: Work-life balance

Related to finding a balance between economic success and wellbeing, career success was also defined as reaching work-life balance. However, work life balance was seen as particularly challenge for women. Women described how having a family was an important factor to consider when discussing career success. Furthermore, starting a family and getting pregnant was considered an obstacle to career success and a challenge that they must face when consider their career choices and how they will measure their career success, as employment conditions are perceived as not compatible with family responsibilities, which particularly affects women’s career choices [78].

Hence, they saw family primarily as an obstacle in this context, considering the challenges of work-life balance, gender stereotypes, and expectations: *“I have come to terms that I am expected to choose between my career and for example having a family*, *which is not something men have to worry about”* (P7; woman; mean SSS). Women expressed their worry about the consequences that having a family would have for their career, as their perceived that to balance their work aspirations with family responsibilities was difficult. Indeed, having a family and its negative impact on future career success was a concern particularly for low SSS, female students. These findings support previous research showing how—for women—marital status can be detrimental for their career success prospects, as they are seen as less productive by their employers, compared to married men [32]. At the same time, the endorsement of more “traditional” values regarding women’s success was discussed when women considered “work-life balance” in relation to success, and family expectations had an important role in conveying these expectations: *“Being from a very conservative family means*, *as a woman*, *I should be married and giving birth to children and not studying*, *so I sadly do not have the financial support from my family here”* (P12; woman; low SSS).

Students’ experiences based on their intersectional identities established the challenging contexts that low SSS women face when pursuing career success. This, in turn, shaped their definitions of career success. Hence, students’ perspectives demonstrate how gender stereotypes about the traditional role of women are still present, especially when intersectional experiences (e.g. gender and social class) are taken into account.

### Lack of clarity about what career success is

A small group of students reported being either unsure or not knowing how to define career success. This uncertainty was mostly shaped by contextual variables, particularly perceived young people’s unemployment. From the perspective of these students, social circumstances were likely to affect employability opportunities and, consequently, make future career success unpredictable. In this way, for those students who perceived an uncertain future, career success was associated with secure employment which, from their perspective, looked difficult to achieve in the current times. Although these perspectives were not associated by students to particular identity experiences, the main focus on financial and job stability might lead us to think that, again, socioeconomic experiences played an important role in the construction of this lack of definition.

### Career success and perceptions of unemployment: Social and contextual constraints

Broader contextual circumstances, particularly the Covid-19 pandemic and perception of unemployment played a critical role in students’ expectations of success and, this in turn, changed how students defined career success. The Covid-19 pandemic has been shown to have widened existing social inequalities [79] and this also was the case here. Students recognised different situations that made it difficult for them to measure and define career success: *“This is currently a difficult question to answer with the pandemic*!*”* (P13; man; high SSS).

Furthermore, for those students from more disadvantaged backgrounds, there was a recognition of greater uncertainty: *“I just feel like the job market is so saturated*, *that employers would go for people with more experience and better grades than me”* (P9; woman; low SSS). Indeed, they recognised that having a degree does not necessarily mean that they will secure better career opportunities: *“Just getting any job in my discipline at this point would be a success to me because I know all of my friends from back home are struggling to get any work related to their degrees”* (P10; woman; mean SSS).

Students also mentioned job market saturation as a challenge to their success in the workforce and in defining their expectations of career success. They referred to examples from their peers and friends that having to work in any job available after they were awarded their degree: *“Having the degree doesn’t really guarantee you a job (…) I know people who have law degrees working in supermarkets*, *but on the other hand I know people that are emploted [sic] straight out of uni”* (P29; man; low SSS). Hence, for a group of students, HE was not necessarily a means to reach career success, especially in the current context of massification of HE (as more people have a degree), and increase of precarious job positions offered [6].

### Unclear definition of career success

A small group of students reported not having a clear idea about what success could look like, nor what their expectations of career success were: *“I wouldn’t say that I have clear career aspirations”* (P23; man; high SSS). This lack of clarity about success encouraged students to engage with ideas about success that do not necessarily make sense to them: *“Hard work is subjective*, *results are objective*. *I still haven’t really understood success to be honest”* (P23; man; high SSS).

Indeed, for these students, the lack of clarity about success was related to a sense of a wider spectrum of potential opportunities and choices. Participants shared different options about their future plans, which made them undecided about their future and how to understand career success: *“I think the future will decide that for me (…)”* (P8; man; mean SSS). Hence, to be able to take time to explore different options without economic and social pressures helps students navigate how they enter the workforce from a different, even privileged, perspective.

## Discussion

In this study, we used a social identity framework to investigate how university students define career success, and if and how their associated these definitions with their gender and SSS social identity-based experiences. Overall, our findings support similar studies conducted with university students about their conceptualisations about career success (e.g. [17,36]). Moreover, our study provides unique and complementary insights around students’ conceptualisations about career success by illustrating the role that students’ gender, SSS, and intersectional experiences can play -from their perspective- in how they define career success. We demonstrate that students report different definitions of career success, and—to differing degrees—explained them in relation to their experiences related to their gender, their SSS, or the intersection of these identities. Thus, our findings contribute to previous research looking at definitions of career success and the role of other intersectional identity experiences (e.g., in terms of gender and ethnicity [32,53]; social class and ethnicity; [54]). Furthermore, to consider the intersection of gender and social class in students’ definitions of career success is important in understanding the role of multiple and intersectional inequalities. Our study provides further evidence to understand career success beyond executive/professional/managerial positions [e.g. 43,44] or specific disciplines (e.g., [36]), showing how individuals outside these roles understand career success and its barriers.

Moreover, previous research has focused on potential demographic differences in individuals’ definitions of success [e.g., 32,81], and our study provides an understanding of how students themselves integrate their identity experiences in their career success definitions. Our study provides insights to understanding career success definitions as built on intersectional experiences between gender and socioeconomic status. Intersectional experiences of disadvantage were emphasised by a group of students -particularly low SSS women-, as they have to navigate family, gender and financial constraints.

These barriers produced particular experiences that shaped how students define career success, interacting to show how intersectional identity experiences are not just a cluster of different inequality experiences-rather they interact to create particular experiences of inequality [68]. For example, what might be a goal for some women (work-life balance) is indeed seen as a challenge to others. The interviews showed how gender norms still have a differential role in how students approach career success, putting more pressure on women, as they feel that they need to ‘have it all” -career success and family- [42], which would be even more difficult for those female students who face economic challenges. Hence, although women in Western society have increased their access to HE and the workforce [80], this might be only the case for a specific group of women: those that have economic resources to facilitate their HE journey and subsequently give them access to the workforce. Thereby, gender equality strategies in HE need to consider women from different socioeconomic backgrounds, looking at how intersectional experiences of gender and social class shape individuals understanding of career success and, therefore, their success expectations.

The current research sheds light on an important paradox in higher education: while students tend to define career success in relatively individualistic ways, such as individual mobility, financial success, or personal development, it was clear that their group memberships (gender, SSS) and related experiences played an important role in creating notable differences in definitions of career success. For example, family beliefs about permeability and success were important, particularly for low SSS students (see [81]). Our findings support previous research showing that when students recognised their family struggles, they define career success as individual mobility and financial achievements and value HE as a tool for improving their future and to not have the same SSS-based experiences as their parents [82]. Moreover, our findings show that the relationship between economy and wellbeing [48] is important in students’ conceptualisations of career success.

Although family plays a relevant role in students career choices, it is important to acknowledge how broader social and economic contexts shape social inequalities [83]. University discourses and practices about the permeability of group boundaries in our society promote the idea that moving from one group to another with more status is possible (see [84]). Universities promote the sense of a permeable and meritocratic system [85], and our findings show how students recognised universities as a tool for achieving career success, which was associated by students with a better quality of life. However, this is problematic as research has shown that meritocratic promises about success have been shown to be ambiguous [86] and false [87]. Therefore, although students enter HE with the purpose of gaining the skills and tools to improve their—and sometimes their family’s—quality of life, it may be the case that these outcomes are not attainable to those that need it most. In this way, students’ definitions of career success show how universities, despite their efforts, might be promoting and reproducing social and economic inequalities, rather than overcoming inequalities.

The failure to acknowledge a broader range of definitions of career success—beyond individual mobility—alongside students’ varied life and educational trajectories, may worsen students’ perception of the possibility of career success when they do not fit the prototypical conceptualisations about career success (e.g., finding a job in a prestigious organisation) or don’t have the resources and opportunities to achieve these goals. In turn, students included what they felt was possible for them as part of their career success definitions, bearing in mind their perceptions of society and intergroup relations (e.g., group permeability), and these conceptualisations of career success may not be recognised by universities. As more options to conceptualise career success are possible and organisations recognise that career success outcomes are not necessarily related to individual effort and motivation, students also have further opportunities to access more diverse and less individualistic parameters to evaluate their own career success, which promotes a sense of less constraints in how they evaluate their career success, ambition and career choices.

### Theoretical and practical implications

Our findings contribute to the understanding of career success and have a number of both theoretical and practical implications. Our study contributes to previous research about how individuals define career success. It also provides support for the idea that conceptualisations of career success are contextual and shaped by identity-based experiences, such as gender and SSS. Students’ expectations of success will be guided by what they think is possible, which is itself shaped by social processes [88,89]. For a similar perspective on success being underpinned by social and contextual processes, see [90]). Therefore, our findings have theoretical implications in how career success is defined in the context of higher education, providing further evidence that career success is a multidimensional concept, and suggesting that success may need to be conceptualised and operationalised through a more social and context-dependent lens.

Our findings also contribute to the current debates about the crisis of the neoliberal model in higher education [91], whereby career success is conceptualised through a highly individualistic lens (e.g., notions of career success being rooted in individual effort and responsibility; [92]), and the lines between individuals’ choices -without taking into account how social context shapes these choices-, and career success seem blurred. The assimilation of individuals’ choices and career success has implications for understanding and reflecting about which ideas of success are being shared in our society and reproduced in HE settings. Our study expands previous research about students’ definitions of career success in educational contexts [17], considering how university organisational discourses, such as widening access/participation might facilitate individuals’ engagement with individual mobility beliefs as a way to reach career success, or with social creativity strategies to boost their group status, rather than social change strategies to challenge the status quo. Therefore, career success is not a neutral nor objective concept and rather, encapsulates social practices and norms reproducing gender and socioeconomic inequalities. This is critical, as students associated career success with different outcomes, such as financial security, happiness, or a better quality of life.

For example, for women, career success definitions and what they see as possible are not homogeneous and present different nuances when we include their socioeconomic social identity. To our knowledge, research on gender differences in career success has not considered fully the role of the intersection of gender and socioeconomic status, focusing mostly on college educated, professional women. Our study provides exploratory results for this vacancy, demonstrating that the concept of gender equality needs to integrate intersectional dimensions, otherwise future work will overlook how, especially for low SSS women, gender equality is still a challenge. Our research provides further support to consider socioeconomic status as an interrelated aspect of gender inequality in HE and in the workplace [93].

Our study provides support to previous research showing that the concept of career success implies nuances that need to be considered [94], and that these nuances can be explained considering an intersectional approach (see [95]). Hence, questions that might appear as simple (“what is career success?”) are actually much more complex than previously thought. The emphasis in using one approach to understand identities and social issues is detrimental in capturing the complexity of these issues. Our research shows that the social identity approach and intersectionality can contribute to each other in complex and heterogeneous contexts [96], such as educational settings. Furthermore, these approaches can benefit from following a bottom-up approach [97], taking into account how participants experience their intersectional identities, especially when they talk about their challenges and barriers to pursuing career success.

These findings also have practical implications. Our study shows that universities need to value different conceptualisations of career success rather than prioritise the ones associated with prestige and/or status (e.g., achieving individual mobility as part of Widening Access programmes), that will be perceived by those belonging to high status groups as more realistic for them and therefore will be an important part of their success conceptualisation, where they may not be for those from low-status groups. Furthermore, universities need to acknowledge how career success outcomes are not just explained by individuals’ effort, work or personal choices. Rather, career success outcomes are explained, at least in part, by social, economic and cultural constraints that benefit to the most privileged groups.

Furthermore, considering multiple career success definitions and the role of social identities in how these definitions are shaped also has practical implications for universities, as to present a range of different ways to understand career success could promote different criteria to understand and evaluate individuals’ success expectations. For example, if universities define career success as individual mobility (e.g., Widening Access programmes), groups that don’t see individual mobility as possible and, in turn, create their definitions about career success without these elements, will be categorised with lower success expectations. Doing this, universities, instead of promoting social change and equality, will keep emphasising outcomes that reproduce social inequities (see [98]). Following these terms, definitions of career success may be considered prescriptive rather than descriptive, as they focus on the conceptualisations of career success of particular groups, and look to apply these as the norm for all individuals, without recognising particular challenges related to equality of access to resources and social opportunities to accomplish one’s goals (for an example of analysis of mis-measures in education, see [99]).

### Limitations and future research

To analyse and evaluate the findings from this study, we must consider its limitations. First, due to the nature of our study, we explored notions of career success in a broad group of students from different universities. Therefore, it was not possible to analyse all the potential nuances when students described success (e.g., including organisational practices that might be different according to discipline or universities). Considering the extensive research about levels of success differences among different disciplines and students from different universities (see [100,101]), future research needs to look at the potential role of disciplines and university status in students’ approaches to career success.

Additionally, our research focused on young adults, and undergraduate students specifically, which means that is an open question as to whether our findings generalise to other student populations. Future research needs to explore how these other groups define career success, especially underrepresented groups in HE contexts, in terms of age (e.g., mature students), education level (e.g., postgraduate students), universities, and field of study (e.g. STEM disciplines).

Furthermore, future research needs to focus on what identity mechanisms underlie students’ definitions of career success, and whether and how this might vary across particular groups. For example, research has shown that the identification with group norms influences students’ definitions of career success [17]. In other words, future research should not only explore *what* career success is for students, but also *why* and *how* they create these conceptualisations.

Third, we explored career success definitions without considering the life stage of participants. Further studies need to acknowledge and explore how success as a construct is understood as a process during time, as previous research has shown that, for example, at the beginning of their career, men and women in different disciplines are equally ambitious, but women’s ambition decreases over time compared to men [56]. One reason for these differences over time is the lack of role models that allow women to see success as possible. Workplace characteristics are therefore critical in promoting the participation of underrepresented groups, such as women and students with low SSS, to convey with practical actions that success can be possible for different individuals. Hence, future research needs to include different methodologies (e.g., qualitative longitudinal studies, narrative inquiry, storytelling, experimental studies) to understand how conceptualisations about career success–and success in general—might change and transform over time and under certain conditions (e.g., educational institution norms, perceived permeability). Likewise, as our study explored students meaning and social identity experiences, further research could include further details about how students express these meaning and experiences, either with analysis looking at students’ verbal expressions or analysis of the recurrence of certain words associated to success.

## Conclusion

In this paper, we have analysed how students define career success and if and how they associated their gender and SSS social identity-based experiences with these definitions, from an intersectional and social identity approach. Although students define success in individualistic terms, especially for students from disadvantaged backgrounds, their social identity-based experiences (gender and SSS) were important to understand their success definitions, as their previous experiences provided background to understanding the reasons underlying their approaches to career success. In this context, intersectional identity experiences were stressed when female students mentioned challenges and barriers perceived to reach success, such as family expectations, gender norms, challenges in work-life balance for women, and anticipated lack of fit in certain institutions. This is critical as the boundaries between career success and individual choices are often difficult to differentiate, putting career success responsibility mostly on the individual, without taking into account social constraints. These findings highlight the importance of considering how the intersection of gender and SSS must be considered to understand students’ conceptualisations about career success, and that gender equality research needs to include an intersectional perspective. Our study also has practical implications, as research needs to consider different success definitions, and universities need to put support in place to facilitate and recognise as valuable all types of success for students, regardless their gender and SSS.

## Supporting information

S1 Table Distribution by gender and subjective social status. (DOCX)

S2 Table Interview script. (DOCX)

S3 Table Initial list of codes. (DOCX)

S4 Table Development of codes, subthemes, and themes. (DOCX)

S1 Data (PDF)
