# Supplementary material for: Gender expectations, socioeconomic inequalities and definitions of career success: A qualitative study with university students
Source: PLoS One. 2023 Feb 24;18(2):e0281967. doi: 10.1371/journal.pone.0281967 (PMC9955979; doi:10.1371/journal.pone.0281967)
Supplement: S1 Table — (DOCX) [file pone.0281967.s001.docx]

**S2 Table 2**. Distribution by gender and subjective social status

| **Subjective social status** | **Gender** | | **Total** |
| --- | --- | --- | --- |
|  | **Women** | **Men** |  |
| **Low** | 6 | 6 | 12 |
| **Mean** | 7 | 3 | 10 |
| **High** | 6 | 8 | 14 |
| **Total** | 19 | 17 | 36 |
