# Supplementary material for: Gender expectations, socioeconomic inequalities and definitions of career success: A qualitative study with university students
Source: PLoS One. 2023 Feb 24;18(2):e0281967. doi: 10.1371/journal.pone.0281967 (PMC9955979; doi:10.1371/journal.pone.0281967)
Supplement: S2 Table — (DOCX) [file pone.0281967.s002.docx]

**Interview Script**

1. How would you define success? What means to you?
2. Regarding your career aspirations, how would you define being successful in your career?
3. What are your expectations after your graduation?
4. Do you think that your university studies will help you to reach the levels of success that you expect? Why?
5. Do you think that how do you feel about your university affect your expectation of success? Why?
6. Do you think that your gender and class have affected your expectations of success?
