# Supplementary material for: Gender expectations, socioeconomic inequalities and definitions of career success: A qualitative study with university students
Source: PLoS One. 2023 Feb 24;18(2):e0281967. doi: 10.1371/journal.pone.0281967 (PMC9955979; doi:10.1371/journal.pone.0281967)
Supplement: S3 Table — (DOCX) [file pone.0281967.s003.docx]

S3 Table 3. Initial list of codes

| First round of codes |
| --- |
| Academic Success & Career Success  Being mentally happy  Being optimistic to success  Brexit  Career choices & Employability  COVID & Employability  Don't have clear career aspirations  Don’t understand success  Expectations of high salary  Family expectations  Family struggles & Motivation to success  Fitting in to success  Further education to not be unemployed  Gender & Work life balance  Graduate Scheme  Having a safe space to success  Having hobbies to reach success  I’m not sure what contributes to success.  Lack of confidence affects success  More success than family [FG]  No expectations of salary  No hope of success [stuck in social position]  No plans after graduation  Parents & Gender expectations  People you live impact success  Pursue further education to success  School shaped university expectations  Social class & Definition of success  Some people don’t worry about success  Success & Always want something bigger  Success & Financially weak family  Success & Personality  Success & Work experience  Success and Unemployment  Success as a high payment job  Success as achieving goals  Success as achieving my goals  Success as applying and having a job ready  Success as being able to live a good life  Success as being at managerial position  Success as being happy  Success as being in a positive mind frame  Success as being my own boss  Success as being proud  Success as being team leader  Success as comfort & decent life  Success as discovering something new  Success as doing moderately well in all areas  Success as feeling confident and secure  Success as financial stability  Success as finding friendly colleagues  Success as forming friendships and relations with colleagues  Success as getting what I want  Success as happy with any job  Success as having a good salary  Success as having a job that enjoy  Success as having a stable job  Success as having good grades  Success as helping normal people  Success as helping people  Success as improvement  Success as learning  Success as lifestyle [upper class]  Success as making a difference  Success as making others happy  Success as meaningful job  Success as motivation to apply learnings  Success as resolve problems at work  Success as setting goals  Success as working in a profession that motivates me  Success as working in interested area  Success as working my way up  Success as working on research  Success Benchmarks  Success expectations changes [ambition]  Success has individual meanings  Success helps to showcase university to future students  Success is achieving goals  Success is enjoying life  Success is just passing  Success is NOT financial  Success is subjective  Success was imposed  Taking time off  University & Motivation to success  University changes definition of success  University employability promises  University satisfaction affects success expectations  Wellbeing to success  World current reality |
