# Supplementary material for: Gender expectations, socioeconomic inequalities and definitions of career success: A qualitative study with university students
Source: PLoS One. 2023 Feb 24;18(2):e0281967. doi: 10.1371/journal.pone.0281967 (PMC9955979; doi:10.1371/journal.pone.0281967)
Supplement: S4 Table — (DOCX) [file pone.0281967.s004.docx]

**S4 Table 4**. Development of codes, subthemes, and themes.

| **Initial codes** | **Final codes** | **Initial subtheme name** | **Second subtheme name** | **Final subtheme name** | **Final theme** |
| --- | --- | --- | --- | --- | --- |
| Success as helping normal people  Success as helping people  Success as making others happy | Improve others people life | Success as meaningful work experiences | Career success as meaningful work experiences: The role of previous socioeconomic experiences | Career success as meaningful work experiences: The role of previous socioeconomic experiences | **Career success as personal development** |
| Success is enjoying life  Success as making a difference  Success as meaningful job | Meaningful experiences |  |  |  |  |
| Success as forming friendships and relations with colleagues  Success as finding friendly colleagues | Workplace relations |  |  |  |  |
| Success as feeling confident and secure  Success as working in a profession that motivates me  Success as working in interested area  Success as being proud  Success as achieving goals  Success as achieving my goals  Success as setting goals  Lack of confidence affects success | Success and personal grow |  |  |  |  |
| Being mentally happy  Success as being happy  Success & Personality  Success as happy with any job | Being happy | Success as positive experiences | Career success as happiness | Career success as happiness |  |
| Being optimistic to success  Having hobbies to reach success  Success as being in a positive mind frame | Positive mindset |  |  |  |  |
| Success as having a job that enjoy | Enjoy work |  |  |  |  |
| Career choices & Employability  Success & Work experience | Career choices | HE as a tool to success (for some) | HE as a tool for career success | HE as a tool for career success | **Career success as individual mobility** |
| University & Motivation to success  University changes definition of success  University employability promises  University satisfaction affects success expectations  Academic and Career success | University shapes success |  |  |  |  |
| Further education to not be unemployed  Graduate Scheme Pursue further education to success | Education as an investment |  |  |  |  |
| Success as a high payment job  Success as financial stability  Success as having a good salary  Expectations of high salary  Success as being able to live a good life  Ambivalent towards money | Financial success | Success as financial security: The role of family | “I did not grow up rich”: Career success as financial security | “I did not grow up rich”: Career success as work success |  |
| Success as having a stable job  Success as applying and having a job ready | Having a stable job |  |  |  |  |
| Family expectations  Family struggles & Motivation to success  More success than family [first gen]  People you live impact success  Success was imposed  Success & Financially weak family | Family expectations |  |  |  |  |
| Success as working my way up  Success as being at managerial position Success as being my own boss  Success as being team leader  Success & Always want something bigger  Success as resolve problems at work | ‘Working my way up’ |  |  |  |  |
| Gender & Work life balance | Success as work life balance | A gendered definition of career success: work-life balance | A gendered definition of career success: work-life balance | A gendered definition of career success: work-life balance |  |
| Parents & Gender expectations | Gender roles and family lack of support |  |  |  |  |
| Brexit  COVID & Employability  World current reality  No hope of success [stuck in social position] | Social context as a source of success instability | Success and perceptions of unemployment | Career success and perceptions of unemployment: Social and contextual constraints | Career success and perceptions of unemployment: Social and contextual constraints | **Lack of clarity about what career success is** |
| Success and Unemployment | Concerns about job market |  |  |  |  |
| Don’t understand success  I’m not sure what contributes to success. | Don’t understand success | Unclear definition of success | Unclear definition of career success | Career success and perceptions of unemployment: Social and contextual constraints |  |
| Don't have clear career aspirations  No expectations of salary  No plans after graduation | No expectations of success |  |  |  |  |
