# Supplementary material for: Gender expectations, socioeconomic inequalities and definitions of career success: A qualitative study with university students
Source: PLoS One. 2023 Feb 24;18(2):e0281967. doi: 10.1371/journal.pone.0281967 (PMC9955979; doi:10.1371/journal.pone.0281967)
Supplement: S1 Data — (PDF) [file pone.0281967.s005.pdf]

Quote	Final code	Final subtheme	Final theme
I haven't given to much thought to be frank (salarys). I would like an income which would allow me to get the necessities and have a comfortable life I guess. If I become which give most of it to good causes. Job interests are many, I'm still undecided, I'm doing sports science but I don't want to work with athletes but rather normal population and use exercise and nutrition to make life better for them	Improve others people lives	Career success as meaningful work experiences: The role of previous socioeconomic experiences	Career success as personal developmen t
For example education. I actively work towards providing children from underprivileged backgrounds the chance at getting a good education from the start, and this can be helped immensely through donations and helping existing organisations who are working at solving this issue.	Improve others people lives	Career success as meaningful work experiences: The role of previous socioeconomic experiences	Career success as personal developmen t
Being in a place, personally, of genuinely helping others	Improve others people lives	Career success as meaningful work experiences: The role of previous socioeconomic experiences	Career success as personal developmen t
I would hate to have a boring desk job and instead I would want to be doing something that is helping people and actually making a change	Improve others people lives	Career success as meaningful work experiences: The role of previous socioeconomic experiences	Career success as personal developmen t

I think being successful in your career is ensuring that you set goals relating to what you want to achieve and seeking to accomplish them. Many people enter a career and wander aimlessly throughout each day for years on end and if asked about their job and whether they have a successful career they can't answer the question as they don't really know what the purpose they have for being there is. For me success in my career would be picking an area that I am passionate about and can see the opportunity to affect change in a local or societal level. I would view success as the ability to improve the lives of other people, compared to the vision some people have of a large bank balance equating to success. If I can change someone's life for the better then that would be the ultimate	Improve others people lives	Career success as meaningful work experiences: The role of previous socioeconomic experiences	Career success as personal development
Being a caring doctor who looks out for the patients' concerns and looks at them as a person.	Improve others people lives	Career success as meaningful work experiences: The role of previous socioeconomic experiences	Career success as personal development

I believe success is taking what you have learned from uni and sharing it with people you meet in the future, e.g. if I become a teacher I will share my experiences and advice as in my high school I did not have a lot of support from my teachers so I was scared to go to uni as I did not know what to expect (...) Just as important as it is for my students to learn it is just as important I learn their culture and share the values of their culture with them.	Improve others people lives	Career success as meaningful work experiences: The role of previous socioeconomic experiences	Career success as personal developmen t
For my career id say I would be succesful if I can help clients (…).	Improve others people lives	Career success as meaningful work experiences: The role of previous socioeconomic experiences	Career success as personal developmen t
Discovering new things and applying it to the society. So to help others with my research. Also, because there is so many false information around nowadays.	Improve others people lives	Career success as meaningful work experiences: The role of previous socioeconomic experiences	Career success as personal developmen t
Ive meandered between different career paths and im thinking of going into health care and helping people who are struggling with health both physically/mentally! I think that would be a successful career is helping people go from a bad to good place and achieve a better version of themselves.	Improve others people lives	Career success as meaningful work experiences: The role of previous socioeconomic experiences	Career success as personal developmen t
Making others happy.	Improve others people lives	Career success as meaningful work experiences: The role of previous socioeconomic experiences	Career success as personal developmen t

That I am making a difference to peoples lives and that I am no longer in debt	meaningful experiences	Career success as meaningful work experiences: The role of previous socioeconomic experiences	Career success as personal developmen t
Success to me means that I have found something which is meaningful. I would consider myself successful if I were pursuing something meaningful and which was generating a decent livelihood for me. If, for example, I had a particular area of law that I felt was profoundly worthwhile studying and becoming an academic for, that is success (at all stages of the career). Success is not pecuniary in nature, but this can come into it.	meaningful experiences	Career success as meaningful work experiences: The role of previous socioeconomic experiences	Career success as personal developmen t
I think for now success is enjoying life and making the most of every second. My final bit of success on the plant when die is knowing that ive left in a better place than I found it!	meaningful experiences	Career success as meaningful work experiences: The role of previous socioeconomic experiences	Career success as personal developmen t
I would also say it includes forming friendships and relationships with your colleagues.	workplace relations	Career success as meaningful work experiences: The role of previous socioeconomic experiences	Career success as personal developmen t
I think by belonging, it is more finding your people (As described before), finding a friendship group and people you enjoy spending time with	workplace relations	Career success as meaningful work experiences: The role of previous socioeconomic experiences	Career success as personal developmen t

I think depending on the work itself, I still expect to find friendly colleagues, as I am a person who values good relationships around me.	workplace relations	Career success as meaningful work experiences: The role of previous socioeconomic experiences	Career success as personal developmen t
Success to me is feeling contented with myself in life, in all areas.	being happy	Career success as meaningful work experiences: The role of previous socioeconomic experiences	Career success as personal developmen t
Success to me is not materialistic such as a big mansion or sports cars. Ultimately success is being happy with what you do or what you already have and fulfilling your goals that you set for yourself.	being happy	Career success as meaningful work experiences: The role of previous socioeconomic experiences	Career success as personal developmen t
I found other friends I have from working class and middle class families put more emphasis on success as being something that makes you feel like you are doing something you enjoy and being content with your life (...) Yeah, more emphasis on loving your job and surrounding yourself with friends you love and family.	being happy	Career success as meaningful work experiences: The role of previous socioeconomic experiences	Career success as personal developmen t
Success to me is doing well and being happy (…) Being successful in my career would be finding a job that I really enjoy and am happy doing whilst working hard to make a difference	being happy	Career success as meaningful work experiences: The role of previous socioeconomic experiences	Career success as personal developmen t

Being happy and confident in what I do (…) Again, being happy and confident in my work. If I'm miserable, I would think I've failed.	being happy	Career success as meaningful work experiences: The role of previous socioeconomic experiences	Career success as personal developmen t
If you are happy with what you have achieved and what you are doing in life then (…) Doing what you enjoy and getting to where you want to be in your career would be a success. For me I would like to specialise in a specific area I think	being happy	Career success as meaningful work experiences: The role of previous socioeconomic experiences	Career success as personal developmen t
finding happiness and comfort (…) Finding a path that makes me happy	being happy	Career success as meaningful work experiences: The role of previous socioeconomic experiences	Career success as personal developmen t
To me being happy at what I am doing is how I would define succes in my career. To me if you are not happy you are not sucessful, even if you have the highest pay job or thst you are well known and famous if you are not happy it is not meaningful and you are not successful In my eyes.	being happy	Career success as meaningful work experiences: The role of previous socioeconomic experiences	Career success as personal developmen t
Success for me means where you are in the point of life where you are happy and content (do not know if that is the right word) with yourself either professionally or personally (...) Having a peaceful life with your loved ones close by and enjoying life even in the little moments is also success for me.	being happy	Career success as meaningful work experiences: The role of previous socioeconomic experiences	Career success as personal developmen t

I don’t think too much about my job after graduation I'm just focusing on getting through vet school and would be happy with any job as a doctor really	being happy	Career success as meaningful work experiences: The role of previous socioeconomic experiences	Career success as personal developmen t
being in a positive mind frame consistently.	positive mindframe	Career success as meaningful work experiences: The role of previous socioeconomic experiences	Career success as personal developmen t
In my career being successful right now is finding a job in the future that I enjoy doing regardless of the payment that I am earning.	having a job I enjoy	Career success as meaningful work experiences: The role of previous socioeconomic experiences	Career success as personal developmen t
I would say it’s a learning experience to know that its not the right job for me and then move onto another job where I was happy, and continue the success by being promoted in the job I like.	having a job I enjoy	Career success as meaningful work experiences: The role of previous socioeconomic experiences	Career success as personal developmen t
If entering the work force, I expect getting a job that I enjoy, with decent pay and opportunity for progression. Implicit in this is that I do expect to have a job after graduation. -	having a job I enjoy	Career success as meaningful work experiences: The role of previous socioeconomic experiences	Career success as personal developmen t
I am still on the hunt for a job, but I would say that to be successful is to do a job that you enjoy and you want to do for the rest of your careerr.	having a job I enjoy	Career success as meaningful work experiences: The role of previous socioeconomic experiences	Career success as personal developmen t

For me, success would mean achieving the dreams I have, it is of course being happy with what I do and working hard towards a goal (which I will hopefully achieve).	personal grow	Career success as meaningful work experiences: The role of previous socioeconomic experiences	Career success as personal developmen t
Success for me is being able to say I achieved something and that the hard work I am most of the time putting in has given me better understanding and knowledge	personal grow	Career success as meaningful work experiences: The role of previous socioeconomic experiences	Career success as personal developmen t
Work success is achieving your goals and doing what you love.	personal grow	Career success as meaningful work experiences: The role of previous socioeconomic experiences	Career success as personal developmen t
Success to me means achieving goals	personal grow	Career success as meaningful work experiences: The role of previous socioeconomic experiences	Career success as personal developmen t
I would say success is being able to achieve the goals I set out for myself.	personal grow	Career success as meaningful work experiences: The role of previous socioeconomic experiences	Career success as personal developmen t
Success for me means meeting my personal achievements – if I set a goal for my self and meet it that to me is being successful	personal grow	Career success as meaningful work experiences: The role of previous socioeconomic experiences	Career success as personal developmen t
Success for me is achieving what you have posed for yourself to do. So to achieve the goals and strivings you have for yourself.	personal grow	Career success as meaningful work experiences: The role of previous socioeconomic experiences	Career success as personal developmen t

The point that you realise that you have managed to make most of your goals come true.	personal grow	Career success as meaningful work experiences: The role of previous socioeconomic experiences	Career success as personal developmen t
Getting ‘what I want’ and where I want to be – I had a goal for myself to get a graduate role in X (...) I looked at what I enjoyed and what was available which is why it was ‘what I wanted’ (...) It wasn’t shared by the university, but theres always a focus on getting a job at X. And it is a graduate role (...) but I know its worth it for my career (...) At the moment, my salary pays no role. At the moment for success I wanted to get a good role to make sure that I can build myself up in accounting to get a better salary when im older. But I don’t think salary and successfulness (For me) is interlinked	personal grow	Career success as meaningful work experiences: The role of previous socioeconomic experiences	Career success as personal developmen t
Success to me would be achieving your goals	personal grow	Career success as meaningful work experiences: The role of previous socioeconomic experiences	Career success as personal developmen t

I think success is achieving the goals you set for yourself. It's important not to get caught up on what other people perceive as success as it's well known that many things have different meanings depending on the individual. I believe success comes in the form of improving yourself, your ability to communicate with others, to work alongside others, but also to feel competent enough that you can rely on your own ability. Success to me is seeing a result from hard work, putting in the effort and achieving a result that reflects that and also acknowledging that sometimes it's necessary to fail before you can be successful and it's ok to fail. The lessons from failure can allow you to have a greater level of success as you have changed your approach and outlook based on analysing how and why you	personal grow	Career success as meaningful work experiences: The role of previous socioeconomic experiences	Career success as personal developmen t
Yes – its also when im proud of myself for something, regardless of personal, academic or professional (…) Yes id say so, if im proud of myself for something, I see it as a reason of my success	personal grow	Career success as meaningful work experiences: The role of previous socioeconomic experiences	Career success as personal developmen t
I think success for me is feeling confident and secure in what you're doing	personal grow	Career success as meaningful work experiences: The role of previous socioeconomic experiences	Career success as personal developmen t
I think setting goals allows you to define your success. Once you achieve a goal you have succeeded, and then you can look towards a new one.	personal grow	Career success as meaningful work experiences: The role of previous socioeconomic experiences	Career success as personal developmen t

to work in a profession that motivates me and stimulates me	personal grow	Career success as meaningful work experiences: The role of previous socioeconomic experiences	Career success as personal developmen t
I am currently considering either a career in the industry or in consultancy	career choices	HE as a tool to success	Career success as individual mobilty
I think after your first job and the start of your career my expectations in regards to success and future jobs will start becoming more reliant on the jobs Ive had and experience rather than my degree.	career choices	HE as a tool to success	Career success as individual mobilty
When I visited the open day for my uni (which was actually my insurance choice on UCAS), they did not stop talking about how they had a real, working law firm inside the law school which gave pro bono advice to the public and that some students could work there. They were really pushing this and the professional side of the course – in hindsight, this should have been an indication that academics weren’t prioritised	university shapes success	HE as a tool to success	Career success as individual mobilty
there is a lot of emphasis put on preparing for a career after study, something I believe is vital to help you understand what is out there and to give you guidance on what may be suited to you.	university shapes success	HE as a tool to success	Career success as individual mobilty

The university is popular and well regarded so I expected a high level of teaching and it would put me in a good position to be employable (...)	university shapes success	HE as a tool to success	Career success as individual mobilty
I've had a generally positive experience, and think I've been successful by my own goals and so from that, I feel that I will take that forward and expect to keep being successful in the future.	university shapes success	HE as a tool to success	Career success as individual mobilty
Before uni, I just thought success would be getting a first, then getting my dream job that pays well and life is sorted for me, all from 3 simple years at uni. But now I have experienced a lot of it, i think success is doing the best I can, again in the fields of my life, like friendships, family and careers. I kinda realised that its not as easy I thought it was, I actually need to work hard.	university shapes success	HE as a tool to success	Career success as individual mobilty
I feel like my studies are making a difference to my understand, my knowledge and helping to reinforce what I want to achieve in future and how I need to go about that. So yes, I think my university studies will help me reach the levels of success I seek to attain as they are enabling me to know more about the line of work I want to build a career upon in the next few years.	education as investment	HE as a tool to success	Career success as individual mobilty

I am very passionate about securing a position after I graduate so that I can earn some money and hopefully complete a Master's or a PhD because I currently will not have the funds to do so. Being successful within my chosen career path will involve getting published on scientific papers and working on stimulating clinical psychological research. It will mean getting invited to conferences on my specialised field and gaining many academic connections and contacts.	education as investment	HE as a tool to success	Career success as individual mobilty
Currently, I plan on working for a couple of years after graduating, before going on to complete a master's degree. I do not know what I want to master in, so that is what I need to decide during my time working.	education as investment	HE as a tool to success	Career success as individual mobilty
Yes, to stand out from the millions of other people who have the same degree as me, considering how little jobs there are for my field.	education as investment	HE as a tool to success	Career success as individual mobilty
I belive it will help me to get a good life and secure a job in wa world where getting a job without a higher or further education is hard. Also it iis the only way I can go for my dream job of becoimg a scientist	education as investment	HE as a tool to success	Career success as individual mobilty

Hopefully, be able to successfully enrol on a graduate scheme somewhere in A and then within a few years purchase a house with my partner (...) They are obviously designed for graduates so they provide you with on-the-job training and also pay more than school leaver programmes. You will also likely be in a team with other graduates which can be useful when starting somewhere new as you will be in the same boat and have something in common.	education as investment	HE as a tool to success	Career success as individual mobilty
I don't believe that you can even enter the field I am hoping for without a degree, its near impossible as it is so academically-based. So, in that respect my studies will certainly help me (...) Taking courses which really help to get people on the right track towards getting to where they want to be.	education as investment	HE as a tool to success	Career success as individual mobilty

I don't really know what I would like to do yet. Maybe take a year out and then go into a postgraduate degree or masters as I can't really do much in my career without further education (...) My degree is quite a broad field and to get a decent job relating to my field I would need to specialise in a certain topic so I don't think I could access the types of job I would want long-term just with my bachelors degree (...) That's also why I think its necessary to do postgraduate studies as to differentiate myself from others. Yes, to stand out from the millions of other people who have the same degree as me, considering how little jobs there are for my field.	education as investment		HE as a tool to success	Career success as individual mobilty
I hope to work and keep studying, and gradually become a health professional. Not clear how just yet.	education as investment		HE as a tool to success	Career success as individual mobilty
Id like to eitheir continue my studies (…) Im also looking into masters in different countries.	education as investment		HE as a tool to success	Career success as individual mobilty

Being able to graduate with a 2:1 is what motivates me, it's what I need to do the master's program I want to do so that's most important at the moment. (...) Get the masters first, but I am thinking of getting a PHD but would want to work before then I think (...) It's hard to get a job in my field at the moment without a masters and so success at university helps me get my degree, which helps me get onto a master's course, which then helps me get a job down the line.	education as investment	HE as a tool to success	Career success as individual mobilty
I think the possibility of doing a masters is quite high since my degree now is quite broad and if I want to go into something as specific as XY I may need to get some more knowledge. I also think that the masters is something that will narrow my pathway and help me decide what I really want to do. (...) I believe that my degree now will help me to get to the next level I need to get to but I may need to further my studies to reach the level of success that I want	education as investment	HE as a tool to success	Career success as individual mobilty
My expectations are to take the Bar training course and become a barrister.	education as investment	HE as a tool to success	Career success as individual mobilty

I would like to go into general mixed practice for a bit and then maybe take time out to specialise in a specific area and work in an animal hospital (...) It depends sometimes you specialise by doing masters/PhD or it could be internships with vets or just general progression in career	education as investment	HE as a tool to success	Career success as individual mobilty
I also believe I would put the notion of a master's degree to the backburner as I believe we are in an era that is crying out for support in the health sector and it's already apparent that it's an area that requires a lot of additional resources and I'd like to get involved with that as soon as possible.	education as investment	HE as a tool to success	Career success as individual mobilty
After medical school there are 2 years of foundation training where you work in the hospital. After foundation you can began your specialisation.	education as investment	HE as a tool to success	Career success as individual mobilty
Well I want to become a doctor. So I suppose after graduation, success in this would mean getting a place on either the training programme by A to train to become a doctor, or getting a place on the master's course to support this (...) I want to study further to be able to train as a doctor.	education as investment	HE as a tool to success	Career success as individual mobilty
I will get a TEFL certificate and immediately apply for the programmes in X for teaching English.	education as investment	HE as a tool to success	Career success as individual mobilty

To have done numerous researches and posted many journals. (...)And I would like the place to be somewhere in X (anywhere around central, so about max 1h of train) and salary at around 25000. (...) Yes, I believe they will. Because they will prepare me with all the skills I need to really pursue my career. (...) I mean, not necessary, but it surely helps. So if success for someone is having a lot of money they do not need education for that. But for myself I think education is key, as without education I wouldn't be able to pursue my career aspirations.	education as investment	HE as a tool to success	Career success as individual mobilty
Also, regarding masters applications there is definitely an advantage for students of a higher "social class", for me there are particular masters I would love to do but cant because it is so expensive and the loan for UK students doesn't cover all of it, meaning I would have to apply for scholarships etc. My peers don't have these issues and I notice they just apply for whatever catches their eye, I find that this also makes me feel out of place when they ask about where I'm applying to etc.	education as investment	HE as a tool to success	Career success as individual mobilty
Will probably need to take out a loan. Ideally I would save enough during a year or couple years out after my undergraduate so that I can pay for it myself.	education as investment	HE as a tool to success	Career success as individual mobilty

The 4 extra years of study could be used to work instead, and that is a huge financial loss. I do consider this aspect, because I have siblings that have yet to enter university, and the faster I become financially independent, the better for my family	education as investment	HE as a tool to success	Career success as individual mobilty
My parents support me a lot financially and obviously that is a big help to my studies and future due to the costs of university, and I guess that helps my mental health too as I don't have to worry so much.	education as investment	HE as a tool to success	Career success as individual mobilty
I'm hoping to do a postgraduate professional course called the L which is required in order to practice as a solicitor. I'm going to have to work full time to pay for this and my living expenses, so I expect it to be very difficult and stressful.	education as investment	HE as a tool to success	Career success as individual mobilty
To go on to do a masters and then figure out where I want to work.	education as investment	HE as a tool to success	Career success as individual mobilty
I know I want to maybe look at PHDs	education as investment	HE as a tool to success	Career success as individual mobilty
I can't imagine ever thinking "wow I'm successful now", I think that in our society now its really easy to get caught up in chasing your dream job/life but then when you get it, wanting something even bigger.	personal grow	Career success as meaningful work experiences: The role of previous socioeconomic experiences	Career success as personal developmen t

Trying to do a job better than last time.	personal grow	Career success as meaningful work experiences: The role of previous socioeconomic experiences	Career success as personal developmen t
Getting a high-paying job, having stability	financial success	“I did not grow up rich”: Career success as financial security	Career success as individual mobilty
Id like to live comfortably. I try to be realistic and be humble, so Id like to save up at first. (…) I definitely think I expect to continue my comfortable lifestyle	financial success	“I did not grow up rich”: Career success as financial security	Career success as individual mobilty
I obviously hope to earn enough to have a comfortable life but I don’t really know what hard figure I could attach to that (...) I think comfortable for me would be something where all my basic needs of food, shelter etc. are met comfortably. And by that I mean that there wouldn’t be a need to live paycheck-to-paycheck to meet basic necessities.	financial success	“I did not grow up rich”: Career success as financial security	Career success as individual mobilty

I would consider myself successful if I were pursuing something meaningful and which was generating a decent livelihood for me (...) I think it would be the case that when I get to a certain point in my career, an increase in pay will only marginally affect my quality of life. For example, once you earn £100,000 a year, getting a pay increase to £200,000 a year is obviously a huge difference numerically but really how is that changing your life; both salaries you can access what you need and usually what you want. (...)	financial success	“I did not grow up rich”: Career success as financial security	Career success as individual mobilty
finding happiness and comfort (…) I want to be able to live comfortably, provide for my family and travel, that would be success for me	financial success	“I did not grow up rich”: Career success as financial security	Career success as individual mobilty
Honestly speaking, I have yet to decide if finance is for me completely, but I a very fond of mathematics, numbers and earning money haha, so those 3 things combined, as well as how huge the industry is, and how it combines all aspects of the world, like any news of the world can have an impact on the markets, so you have to be on top of everything.<br>How is being fond of earning money?<br>That industry compensates quite well.	financial success	“I did not grow up rich”: Career success as financial security	Career success as individual mobilty

I think this area has a quite large choice when it comes to jobs. Although it could be very competitive, salaries are quite high.	financial success	"I did not grow up rich": Career success as financial security	Career success as individual mobilty
I loved my field degree at A level, and I researched what degrees have the highest graduate employment rate with highest pay.	financial success	"I did not grow up rich": Career success as financial security	Career success as individual mobilty
I measure success on a big mixture of happiness and money with a big weight on happiness – but money certainly will ease those worries won't it.	financial success	"I did not grow up rich": Career success as financial security	Career success as individual mobilty
In my career, success for me would be defined by earning a good amount of money, so that I can help the wider community outside and lead a comfortable life as well	financial success	"I did not grow up rich": Career success as financial security	Career success as individual mobilty
I expect a great workplace and a very competitive salary.	financial success	"I did not grow up rich": Career success as financial security	Career success as individual mobilty
Yes, I hope to earn at least 25000£, even if that means working outside my area of study. And have peace from the financial point of view.	financial success	"I did not grow up rich": Career success as financial security	Career success as individual mobilty
I want to do something that pays well, obviously, but also not at a sacrifice to my mental health or time. For example if I had a job that paid extremely well but I was unhappy and never had any time to myself, I wouldn't really count that as a win – a success.	financial success	"I did not grow up rich": Career success as financial security	Career success as individual mobilty
Success to me is doing well and being happy	financial success	"I did not grow up rich": Career success as financial security	Career success as individual mobilty

Career success to me is achieving a level of stability in your finances. It is also important to enjoy and find fulfilment in your career.	financial success	“I did not grow up rich”: Career success as financial security	Career success as individual mobilty
pays well enough and enables me to travel with plenty of opportunities to expand and branch out from it	financial success	“I did not grow up rich”: Career success as financial security	Career success as individual mobilty
I think that achieving a decent salary would be a major success, no matter what career I get into. That, and a decent work-life balance	financial success	“I did not grow up rich”: Career success as financial security	Career success as individual mobilty
To me succes means that I am able to live a good life. What I mean by that is that I have got a roof over my head, food and water and I have enough money that I don`t need to worry exessively over what is to come or that I need to be constanly worried over how I am able to survive the next day.	financial success	“I did not grow up rich”: Career success as financial security	Career success as individual mobilty
So being successful can be having a stable job that you do but don’t necessarily feel fulfilled by, but that enables you to pursue your hobbies for example	having a stable job	“I did not grow up rich”: Career success as financial security	Career success as individual mobilty
Well hopefully between now and then I can successfully apply and have a job ready.	having a stable job	“I did not grow up rich”: Career success as financial security	Career success as individual mobilty
But if not, I would like to carry on studying towards a master's degree.	education as investment	HE as a tool to success	Career success as individual mobilty
Ideally id want a job, but im not sure about further study (…) At the start any job but within a year id want to work in my career	having a stable job	“I did not grow up rich”: Career success as financial security	Career success as individual mobilty

To find a job and build up my experience.	having a stable job	“I did not grow up rich”: Career success as financial security	Career success as individual mobilty
I have been interested in human behaviour since a very young age and I wish to apply this knowledge to hopefully fulfill my dream to become a lecturer	career choices	HE as a tool to success	Career success as individual mobilty
I have always enjoyed computers, and wanted to computer science at University to broaden my skills and knowledge.	having a job I enjoy	Career success as meaningful work experiences: The role of previous socioeconomic experiences	Career success as personal developmen t
I wanted to study why people did thing when I was young I would always ask my mum why did people do that or what motivated them to do that. When kept asking those questions my mum and me would read books or watch doucmuntires on these types of topics .	having a job I enjoy	Career success as meaningful work experiences: The role of previous socioeconomic experiences	Career success as personal developmen t
At school I was very into sport, I decided I wanted to learn more about the science and the performance aspect also the benefits aspect of participating or performing at a high level of sport/exercise.	having a job I enjoy	Career success as meaningful work experiences: The role of previous socioeconomic experiences	Career success as personal developmen t
I think that the subject focuses on using math and critical thinking to understand the natural world, and constantly strives to get people to ask questions about why things happen. -	having a job I enjoy	Career success as meaningful work experiences: The role of previous socioeconomic experiences	Career success as personal developmen t

I would like to become a barrister to pursue my love for advocacy and social justice.	Improve others people lives	Career success as meaningful work experiences: The role of previous socioeconomic experiences	Career success as personal developmen t
I enjoy learning more about the business and what I could do to improve what we do. The degree helps me improve upon existing skills.	Improve others people lives	Career success as meaningful work experiences: The role of previous socioeconomic experiences	Career success as personal developmen t
Further, it also means coming from a background that is not supportive, coming from a financially weak family in general and nonetheless making it out of there successfully.	family expectations	"I did not grow up rich": Career success as financial security	Career success as individual mobilty
For some people it might be because of expectations from family, for others they might have gotten some good experience in the field so they have a clearer idea of what they want to do, or for jobs such as in psychology or nursing I imagine the personal aspect helps people to know they want to go into it	family expectations	"I did not grow up rich": Career success as financial security	Career success as individual mobilty
and this does also make me feel a little less motivated as I do not have this financial stability and so I don't feel like I could choose any career to be successful in, and rather I have to find something within stem as this is likely to be higher paying, which I suppose could be more important to me if only because of family life	financial success	"I did not grow up rich": Career success as financial security	Career success as individual mobilty

I will hopefully be able to take the analysis skills I have learnt and apply them into the real world once graduated to help improve the lives of those around us	Improve others people lives	Career success as meaningful work experiences: The role of previous socioeconomic experiences	Career success as personal developmen t
I have always been interested in science and maths and I feel like medicine was the way to use my knowledge to directly affect people's lives. (...) People could be going through issues with work and family do to a disease or disorder and helping them for the better brings me great satisfaction. (...) I want to help people and become a doctor. This is the way to that so that's what really motivates me.	Improve others people lives	Career success as meaningful work experiences: The role of previous socioeconomic experiences	Career success as personal developmen t
I think it was a mixture of reasons, I wanted to study law origionally but I realised I wanted to help people who genuinley needed it, I know that mental health is becoming regarded in recent years as just as important as physical health	Improve others people lives	Career success as meaningful work experiences: The role of previous socioeconomic experiences	Career success as personal developmen t
A mixture, myself, because I need to find it interesting and the organisation because if they don't treat employees well then I wouldn't be happy there. I also think it depends who you work with, people at the same level, if you get on, I think it gives a better feeling of success.	workplace relations	Career success as meaningful work experiences: The role of previous socioeconomic experiences	Career success as personal developmen t

I guess I never really had a chance to really think what success meant to me, or was never able to self define it, it was always something imposed on me and I accepted that	family expectations	“I did not grow up rich”: Career success as financial security	Career success as individual mobilty
In some way yes, but I also think she [family member] wants me to progress in health care as she didn’t until her late 40s. And she has said she wished she done it earlier. So being there helps towards that. (...) Because when I started my university studies, we didn’t think I would carry it on and actually find it too hard to stick to. (My college journey was not very stable) she has told me before she wishes she progressed earlier.	family expectations	“I did not grow up rich”: Career success as financial security	Career success as individual mobilty
Both of my parents have gone through their fair share of bad experiences growing up and that means they’ve ended up working bad jobs with little room for growth, they always said growing up that they wanted us to become the best we can be so we didn’t end up like them	family expectations	“I did not grow up rich”: Career success as financial security	Career success as individual mobilty

A lot of the kids I went to school with were pretty upper-class and so are my grandparents and their ideas of success always felt like it was more closely linked to the sort of lifestyle you could afford, where I found other friends I have from working class and middle class families put more emphasis on success as being something that makes you feel like you are doing something you enjoy and being content with your life.	financial success	"I did not grow up rich": Career success as financial security	Career success as individual mobilty
Its mainly just realising what sort of lifestyle I want to live and realising I have to do a lot of work to get there, and in doing so I need to grow as an idividual	financial success	"I did not grow up rich": Career success as financial security	Career success as individual mobilty
When I was younger my parents actually moved from X to here and Ive been here a very long time. Ive seen how hard they work and even though my parents both have good university degrees and my dad even has a masters, this isnt recognised in this country. So that means they've had to work manual or low paid jobs for most of their lives and I think looking at them and seeing this, Ive always really strived to work hard and study hard to build as good of a life as possible	family expectations	"I did not grow up rich": Career success as financial security	Career success as individual mobilty

I'll be 26 and hopefully no longer a carer, but either a team leader or if something else comes up. However I also think itd be nice to focus like a top up year in something specialised but who knows by that time!	working my way up	"I did not grow up rich": Career success as financial security	Career success as individual mobilty
I would want to get a job related to my area of interest which is developmental disorders and so anything that I could use to further my education or expand career options for that would be seen as a success for me.	personal grow	Career success as meaningful work experiences: The role of previous socioeconomic experiences	Career success as personal developmen t
I think the definition of successful in this case changes over time, like starting off just getting a job and getting your foot in the door would be considered successful to me, and from then it's about getting a better position or becoming more senior, and late on in the career it's about being in a good position to retire.	personal grow	Career success as meaningful work experiences: The role of previous socioeconomic experiences	Career success as personal developmen t
I wont prioritise money greatly over my social life/mental health but I feel like it is a big thing because everybody wants to earn money, right. Like we don't go to uni, which is optional, and spend 2 years doing countless exams and have so much student debt to not be earning money once we get jobs	education as investment	hE as a tool to success	Career success as individual mobilty

Potentially, yes. I didn’t grow up ‘rich’, but I am working harder in my education to be able to be comfortable. Although saying this, I still don’t think having the salary is part of the success that I feel, but it more effects how I work hard because I want to be in a better financial position.	financial success	“I did not grow up rich”: Career success as financial security	Career success as individual mobilty
However if I don’t manage to do that I would really want a managerial position of some kind and I think then I would also feel successful in my career. I guess it is also being able to live a life in which you are happy and comfortable in.	working my way up	“I did not grow up rich”: Career success as financial security	Career success as individual mobilty
I think, in regards to my career, I define success in various stages. The first being getting a job, in general, in a field of my interest and doing a good job. Then, after that, success would be continuously working my way “up” until, hopefully one day, I’d achieve my own ultimate goal of being a lead researcher in a study.	working my way up	“I did not grow up rich”: Career success as financial security	Career success as individual mobilty
For me success in that manner would mean when I would have my own small business and being my own boss.(...) Yes, it is my plan after graduation after doing a master. Not sure what kind of business I would have but something that makes people lives easier and more joyful and not just offer another materialist product.	working my way up	“I did not grow up rich”: Career success as financial security	Career success as individual mobilty

My dad initially wanted me to study business but I did not have an interest so at first it was a bit difficult to understand what I am studying but once he knew I was happy studying it he was happy for me too.	Gender roles and family lack of support	A gendered definition of career success: work-life balance	Career success as individual mobilty
families are often more equal now with women more interested in working and not just staying home to take care of the family. As well as that, families are often quite equal with responsibilities being shared, which I hope carries on.	Gender roles and family lack of support	A gendered definition of career success: work-life balance	Career success as individual mobilty
I also think there has to be a balance between work and life and this leads to successful people as those who are completely consumed by their work are not always happy. I would want a career where I can stop and settle down with a family, but always knowing the job Is still there and possibly going back to it when I can (...) My dream job would be a scientist however I know with the hours and unpredictability it is not always the easiest with a family. (...) Since I love science I would definitely considering going into a less 'hands-on' speciality for example psychology after I have settled down (...) Having a job that is so time consuming will definitely put a strain on relationships and I would always want to be present when my kids are growing up. I was fortunate in	Gender roles and family lack of support	A gendered definition of career success: work-life balance	Career success as individual mobilty

Money is necessary to live on, but I don’t think we should live for money. (...) I feel like my business was earning me money and maybe by society’s standard was successful, but I didn’t feel like a success as I didn’t feel like what I was doing was making a difference to anyone.	financial success	HE as a tool to success	Career success as individual mobilty
I guess it all depends on what people value. I personally know that money is important but I wouldn’t say it’s the thing that drives me or makes me want to be successful, and even though it’s important in life I wouldn’t say it’s necessarily important to me so long as I have enough to live.	financial success	HE as a tool to success	Career success as individual mobilty
I'm not into being rich or making loads of money its doesn’t inspire me that much. I haven't given to much thought to be frank (salarys). I would like an income which would allow me to get the necessities and have a comfortable life I guess. If I become which give most of it to good causes. Job interests are many, I'm still undecided, I'm doing sports science but I don’t want to work with athletes but rather normal population and use exercise and nutrition to make life better for them.	financial success	HE as a tool to success	Career success as individual mobilty

Well ideally I would be working as a scientist assistant, or something similar in a health organisation or hospital, somewhere in such a structure. But if it turns out to be hard to get in, to secure a place I am happy with, I should have a plan b, to keep me going. I am aware also of the need of mental health professionals especially after this pandemic, from the individual level to the organisational and societal levels	perceptions of unemployment	Career success and perceptions of unemployment: Social and contextual constraints	Lack of clarity about what career success is
I used to have a different definition of success, before this experience of university studies in a pandemic, it was more like thriving, being the best version of myself and inspiring and helping other attain that for themselves.	perceptions of unemployment	Career success and perceptions of unemployment: Social and contextual constraints	Lack of clarity about what career success is
I think right now especially, my expectations in terms of the job market are quite low and almost deflated. I think it is already quite difficult to find a job usually but given the pandemic right now it adds extra pressure.	perceptions of unemployment	Career success and perceptions of unemployment: Social and contextual constraints	Lack of clarity about what career success is

I'd say go on into a graduate job, hopefully something in my field of interest. However, given the current situation I am not quite sure yet, as the jobs are limited. (...) So as far as I am aware at least and from what many lecturers talked to us about, the covid-19 pandemic has affected the amount of jobs available. (...) I think that a lot of studies might have had problems in being conducted due to the pandemic at the moment, funding might have been an issue too, so right now, positions in such studies are extremely limited. I hope that we can overcome the pandemic with the vaccine and that going back to normal will also mean more studies starting again in areas I am interested in and that I could then apply for assistant research jobs in these studies.	perceptions of unemployment	Career success and perceptions of unemployment: Social and contextual constraints	Lack of clarity about what career success is
I am not sure due to current circumstances. But, after graduation I would love to do something that pays my bills and I love.	perceptions of unemployment	Career success and perceptions of unemployment: Social and contextual constraints	Lack of clarity about what career success is
This year has been somewhat different in the sense that I haven't been working full time as I lost my job during the pandemic.	perceptions of unemployment	Career success and perceptions of unemployment: Social and contextual constraints	Lack of clarity about what career success is
It makes me feel like I am stuck in my social position and there is no hope of success and I am bound to a low-income menial job forever – which may not be how it goes but it is how the University makes me feel about my own success – even if I end up with a first.	perceptions of unemployment	Career success and perceptions of unemployment: Social and contextual constraints	Lack of clarity about what career success is

The problems I am having at the moment centre around employability for after I graduate which I have received very little aid with	perceptions of unemployment	Career success and perceptions of unemployment: Social and contextual constraints	Lack of clarity about what career success is
Im not sure, it seems like its getting harder and harder for young people out of university to go into the jobs they want and you hear a lot about people with degrees working random jobs that don’t pay very well	perceptions of unemployment	Career success and perceptions of unemployment: Social and contextual constraints	Lack of clarity about what career success is
Currently, from my friends experiences of finding jobs, I do expect the job market to be tough and jobs to be rare to find. (…) Sadly, this is the reality that I have to face. I have to just take it on and try my best to find a job I desire.	perceptions of unemployment	Career success and perceptions of unemployment: Social and contextual constraints	Lack of clarity about what career success is
I expect not to find a job immediately. I think a lot of uni students (including my roommates) think the same, but I wouldn’t do a masters/PhD immediately. (...) I think I would get a full time job in something like retail or food since I have experience, so I’d do the job while looking for a uni career. I expect to get a uni career job within 2 years and if that much time haas passed without me finding anything, I would probably find some experience, while maintaining a part-time job in retail/food.(...)I just feel like the job market is so saturated, that employers would go for people with more experience and better grades than me.	perceptions of unemployment	Career success and perceptions of unemployment: Social and contextual constraints	Lack of clarity about what career success is

Just getting any job in my area at this point would be a success to me because I know all of my friends from back home are struggling to get any work related to their degrees. (...) It's hard to get a job in my area at the moment without a masters and so success at university helps me get my degree, which helps me get onto a master's course, which then helps me get a job down the line.	perceptions of unemployment	Career success and perceptions of unemployment: Social and contextual constraints	Lack of clarity about what career success is
I think the biggest challenge is securing a graduate job given the current climate for young workers. Employability and the skills required for that are not perhaps as much of focus as they should be securing a graduate job related to their degree is a major concern for students everywhere.	perceptions of unemployment	Career success and perceptions of unemployment: Social and contextual constraints	Lack of clarity about what career success is
I don't have many expectations as I don't know what the world will be like, but I don't expect it to be easy to find a career path I am happy with although I am confident I will	perceptions of unemployment	Career success and perceptions of unemployment: Social and contextual constraints	Lack of clarity about what career success is

I think I might struggle for a while finding a job. Ive seen a lot of my older friends getting out of uni and having to work in retail, having the degree doesn’t really guarantee you a job. I think its an individual problem, I know people who have law degrees working in tesco, but on the other hand I know people that are emploted straight out of uni. As well as that I know quite a few middle ages people with degrees working “bad” jobs What do you mean by "bad" jobs? Shop work, cleaning jobs etc.	perceptions of unemployment	Career success and perceptions of unemployment: Social and contextual constraints	Lack of clarity about what career success is
Before Covid-19 my dream was to become a X, that is on pause right now, maybe I can combine both of them having a small business but I suspect that for the next 2 or 3 years that would be a little risky to do. I am still in the process of figuring everything out but I know for sure I want to be my own boss. Yes, Covid for sure forced me a little bit to change direction and yes, I had the business plan in mind before starting my degree and thought it was one way of being my own boss.	perceptions of unemployment	Career success and perceptions of unemployment: Social and contextual constraints	Lack of clarity about what career success is
I think the future will decide that for me, once I find the job that I enjoy, I will look back at the journey that brought me to that situation	unclear definitions of success	unclear definitions of success	Lack of clarity about what career success is

I havent really thought about it too much, I don't know whether ill be staying in this city or moving back home,	unclear definitions of success	unclear definitions of success	Lack of clarity about what career success is
I think some people are more likely to be able to be driven to a point where they just do the best they can and don't worry too much about expectation of success.	unclear definitions of success	unclear definitions of success	Lack of clarity about what career success is
At the moment, my salary pays no role. At the moment for success I wanted to get a good role to make sure that I can build myself up in accounting to get a better salary when im older. But I don't think salary and successfulness (For me) is interlinked	unclear definitions of success	unclear definitions of success	Lack of clarity about what career success is
Can't say that I do, I haven't thought a lot about what area, but possibly I would look to go into teaching – in terms of salary I have no idea	unclear definitions of success	unclear definitions of success	Lack of clarity about what career success is
I wouldn't say that I have clear career aspirations	unclear definitions of success	unclear definitions of success	Lack of clarity about what career success is
"Hard work is subjective, results are objective" I still haven't really understood success to be honest.	unclear definitions of success	unclear definitions of success	Lack of clarity about what career success is
I have to stay with them at my city for at least X years, but my current plan is to move to X after X years as they have a scheme where you can move to different offices after you are fully qualified	education as investment	HE as a tool to success	Career success as individual mobilty

Your mannerisms and personality are defeintely a factor to success. You may really work hard a student, but if you come across as arrogant/ rude, people wouldn't want to work with you. Success often involves an aspect of team work at some point.	workplace relations	Success as meaningful work experiences	Career success as personal developmen t
My career aspirations revolve around research. To me, being successful would be creating an understanding of a previously unknown mechanism. Discovering something or just contributing to part of a complex mechanism would still require determining a previously unknown pathway.	meaningful experiences	Success as meaningful work experiences	Career success as personal developmen t
Wanting to better my career options and to make family proud of me.	family expectations	"I did not grow up rich": Career success as financial security	Career success as individual mobilty
I believe my views about success are more finding my own success spot, not what other people expect or want. And society has so many views and opinions about what others should be doing that they don't think what is success for them.	meaningful experiences	Success as meaningful work experiences	Career success as personal developmen t
The way a person wants to apply what they have learnt. Not mattering on where it has come from.	meaningful experiences	Success as meaningful work experiences	Career success as personal developmen t

Success to me would be doing moderately well in all fields (family, friends and academics), rather than sacrificing one field to go really high in another (e.g. doing really well in my academics, but sacrificing friendships and social life). So I would say success is doing well, but also doing well in all areas of my life. I think being successful in my career is again about balance.	gender roles and family lack of support	A gendered definition of career success: work-life balance	Career success as individual mobilty
I think success is achieving whatever your own personal goal might be. In my case, it would be to successfully complete my undergrad and find a job in X that makes me happy and challenges me at the same time. It means beating the odds to me.	being happy	Success as meaningful work experiences	Career success as personal developmen t
This would then be followed by being able to produce impactful work, e.g. research that is impactful in the particular area if I pursue academia, or if in industry, something that clients can use, or that impacts the organisation.-	meaningful experiences	Success as meaningful work experiences	Career success as personal developmen t
After graduation, I expect to get a role in the research environment. And from their, I would propel myself into the world. I know it will be hard at first but after taking a few years to understand real-life work scenarios, I can accomplish something.	working my way up	"I did not grow up rich": Career success as financial security	Career success as individual mobilty

Success to me is learning new ways of thinking, gaining knowledge of my subject area and developing skills for the future.	meaningful experiences	Success as meaningful work experiences	Career success as personal developmen t
In terms of an expected job, I definitely would think that something where no two days are the same and having the opportunity to solve different problems would constitute a successful job for me	meaningful experiences	Success as meaningful work experiences	Career success as personal developmen t
I was interested in X because I heard a lot of great things about it in terms of applying for jobs after but I always knew I wouldn't want to do pure economics.	improve others people lives	Career success as meaningful work experiences: The role of previous socioeconomic experiences	Career success as personal developmen t
I have a disabled sister and I wanted to be able to work with children like her in a clinical or educational setting (…)	improve others people lives	Career success as meaningful work experiences: The role of previous socioeconomic experiences	Career success as personal developmen t
I have always wanted to be a vet and love animals	improve others people lives	Career success as meaningful work experiences: The role of previous socioeconomic experiences	Career success as personal developmen t
I wanted to be able to provide people with the help I wished id had	improve others people lives	Career success as meaningful work experiences: The role of previous socioeconomic experiences	Career success as personal developmen t

From my experiences yes. Because I am the only one in my family close to getting their University degree and in comparison to my mum who does not have a high school diploma is a bit of a struggle in building a career thus not achieving her goals in mind.	family expectations	“I did not grow up rich”: Career success as financial security	Career success as individual mobilty
I believe a good attitude is very important. You have to be positive, optimistic and respect. Discipline is a major factor because discipline helps you keep your eyes on the prize and focus on tasks. Determination helps you not to throw in the towel when things get difficult. Last focus helps you to be less distracted	enjoyment	Success as meaningful work experiences	Career success as personal developmen t
I believe the environment I’m living in, my health and wellbeing. Also my health and wellbeing will relate as if I am not well, I struggle to motivate myself.	enjoyment	Success as meaningful work experiences	Career success as personal developmen t
Such as work experience, being invovled in other projects outside of education, and taking part in activities and being mentally happy.	being happy	Success as meaningful work experiences	Career success as personal developmen t
If you constantly assume you aren’t good enough and can’t do something, then you will lack motivation, lack self-belief and lack confidence to really push yourself to the limit to see what you can achieve.	personal grow	Career success as meaningful work experiences: The role of previous socioeconomic experiences	Career success as personal developmen t

I feel unsure of myself, if I will be fully prepared for the world after me graduating, a world that has and is fundamentally changing as we speak.	personal grow	Career success as meaningful work experiences: The role of previous socioeconomic experiences	Career success as personal developmen t
I am not 100% sure but I might go on a placement at a hospital and start my foundation programme. How would you reach your aspirations? I would need the proper training which I'll get at uni and during my further training years. This will hopefully build my knowledge and shape me to be a better doctor.	education as investment	HE as a tool to success	Career success as individual mobilty
If pursuing further studies, I expect to get into a good PhD programme, that will at the very least have tuition fees and living expenses covered. I intend to apply for PhD programmes as well as graduate roles, and see who is willing to accept me, before making that decision. I don't think there is a point in thinking about this now, but rather just have a general idea for when I do have to make that decision. But assuming all goes well and I actually have a choice, I think my decision would depend on how much I like the work/field of research, and whether it would be financially okay with pursuing further studies/ whether I think that particular field will benefit me after 3-4 years of extra study.	education as investment	HE as a tool to success	Career success as individual mobilty

Hopefully I would like to continue to study into my masters and then hopefully into my doctorate. Then I would like to go into X.	education as investment	HE as a tool to success	Career success as individual mobilty
(...) I would like to pursue a career in research which is almost impossible without a higher education. But also I believe there is much knowledge you acquire at uni that you wouldn't without studying that higher education is going to be ever more important in the future.	education as investment	HE as a tool to success	Career success as individual mobilty
At the moment I have applied for a masters program which I hope to get into (both because I enjoy what I study but I guess also to not go into the job market just yet).	education as investment	HE as a tool to success	Career success as individual mobilty
I have open mindset as I understand the world is in a strange place right now.	social context as a source of success instability	Career success and perceptions of unemployment: Social and contextual constraints	Lack of clarity about what career success is
This is currently a difficult question to answer with the pandemic! Prior to the pandemics there were far more internships available for me to use as a guage for whether or not I wished to work in a particular industry.	social context as a source of success instability	Career success and perceptions of unemployment: Social and contextual constraints	Lack of clarity about what career success is
I mean skills that increase your chances of being employed so things like training for interviews, assessment centres and CV coaching etc. While some these services are available they are hard access or non-existent.	social context as a source of success instability	Career success and perceptions of unemployment: Social and contextual constraints	Lack of clarity about what career success is

But also money wise, that is a big challenge with the covid situation and the job market, as I am receiving student finance as a migrant worker.	social context as a source of success instability	Career success and perceptions of unemployment: Social and contextual constraints	Lack of clarity about what career success is
I refer more to brexit, especially when it comes to working abroad which I might like to do	social context as a source of success instability	Career success and perceptions of unemployment: Social and contextual constraints	Lack of clarity about what career success is
Currently, from my friends experiences of finding jobs, I do expect the job market to be tough and jobs to be rare to find. Sadly, this is the reality that I have to face. I have to just take it on and try my best to find a job I desire.	concerns about the job market	Career success and perceptions of unemployment: Social and contextual constraints	Lack of clarity about what career success is
Just getting any job in my area at this point would be a success to me because I know all of my friends from back home are struggling to get any work related to their degrees. It's hard to get a job in my area at the moment without a masters and so success at university helps me get my degree, which helps me get onto a master's course, which then helps me get a job down the line.	concerns about the job market	Career success and perceptions of unemployment: Social and contextual constraints	Lack of clarity about what career success is
I think the biggest challenge is securing a graduate job given the current climate for young workers. Employability and the skills required for that are not perhaps as much of focus as they should be, securing a graduate job related to their degree is a major concern for students everywhere.	concerns about the job market	Career success and perceptions of unemployment: Social and contextual constraints	Lack of clarity about what career success is

I have come to terms that I am expected to choose between my career and for example having a family, which is not something men have to worry about.	Gender roles and family lack of support	A gendered definition of career success: work-life balance	Career success as individual mobilty
I guess I am also thinking of the future, because my course is four years long, what if me and my partner decided to start a family, would I be able to continue with my studies. If I did continue, I feel there would be a lot more stress and worry (only if this situation occurred). If certain situations were to happen in the future whilst still studying, I feel as though I would have let my employer down if I couldn't continue studying. I would want to complete my degree; however, I don't know anyone that has completed a degree whilst starting a family. Some females as I mentioned before, might worry about succeeding at university, if they were to become pregnant. I don't see any issues with males and females both having success.	Gender roles and family lack of support	A gendered definition of career success: work-life balance	Career success as individual mobilty
Yes defintely, my university experience has given me countless soft skills, which are essential in any field or industry. Furthermore, the course teaches me a way to think, and solve problems, which will be invaluable in the future.	education as investment	HE as a tool to success	Career success as individual mobilty

This obviously could be due to the technical knowledge you gain but also because of the experiences you accrue over that period.	education as investment	HE as a tool to success	Career success as individual mobilty
It will help get me into a better career which will hopefully lead to that feeling of success. This is due to employers looking for a degree on your Do you think university will help you for your succes plans? Yes, because some people on my course have gained promotions at work due to what they have learnt at university and brought into the work place.	education as investment	HE as a tool to success	Career success as individual mobilty
Yes, since my course involves the basis of understanding that any role in this field would require. They also have many further support roles available alongside to help you I find a job.	education as investment	HE as a tool to success	Career success as individual mobilty
Having qualifications help to find a job and to be successul in life because without qualificaitons, it would be so much harder to find a job.	education as investment	HE as a tool to success	Career success as individual mobilty
I am content with what I learned at university and I am going into the same field as my degree which has undoubtedly helped	education as investment	HE as a tool to success	Career success as individual mobilty
Yes, I believe they will. Because they will prepare me with all the skills I need to really pursue my career.	education as investment	HE as a tool to success	Career success as individual mobilty
I think it definitely puts things into perspective. I now know better than to expect the perfect job straight out of university.	education as investment	HE as a tool to success	Career success as individual mobilty

I think education is the key for me personally, but for other people you can have jobs / success that don't require education. For me, I think its because I want careers in the scientific field, where you need experience, that you can only gain from university. Apprenticeships for example wouldn't give me the qualifications and experience I need	education as investment	HE as a tool to success	Career success as individual mobilty
I do think they will help. Firstly and most simply, because for what I consider to by my goals, I require a degree. So, of course, studying for a degree is a great first step towards success (…)	education as investment	HE as a tool to success	Career success as individual mobilty
I think beside the technical knowledge I would have gained, the transferrable skills that are a part of the degree will hopefully prepare me well for a career in whichever field I wish.	education as investment	HE as a tool to success	Career success as individual mobilty
I think for degrees like mine, whereas in something like medicine all of the learned content (I assume) is useful to the field, for the most part the point of getting a degree like in mathematics is to show that you're capable of learning these things even though the actual content isn't likely to be used again unless you're in research or doing something specific such as statistics	education as investment	HE as a tool to success	Career success as individual mobilty

I think the more knowledge you gain from education, the more opportunities you can have. If you have a good foundation of knowledge to work with, you can choose what you enjoy and chase that opportunity in a certain area. You can apply for so many opportunities because you meet the requirements for them.	education as investment	HE as a tool to success	Career success as individual mobilty
I think I've learnt a lot and my university studies will definitely help me throughout life, and especially through my job as it's in a related field. I'm sure it would be helpful to reach success but I don't think it would probably be the defining factor	education as investment	HE as a tool to success	Career success as individual mobilty
It depends on what you aspire to be and what you want to do with your life – for me, where I want to be and how I value success, I do think education is key (…)	education as investment	HE as a tool to success	Career success as individual mobilty
(...) for myself I think education is key, as without education I wouldn't be able to pursue my career aspirations.Because I would like to pursue a career in research which is almost impossible without a higher education. But also I believe there is much knowledge you acquire at uni that you wouldn't without studying.	education as investment	HE as a tool to success	Career success as individual mobilty

Education is certainly important and I feel it contributes to success within business and in the employability industry. But for myself I think within what I want to do as a job education will be important as a gateway to my success within that field – but not within my success within my happiness	education as investment	HE as a tool to success	Career success as individual mobilty
I think partly, in the sense that without education it is very difficult to be successful, and it is something which needs to be done. However, I do not think it is the one and only key, as there are many other factors to consider, and experiences to be involved with.	education as investment	HE as a tool to success	Career success as individual mobilty
Getting good grades opens new perspective, some universities require a first-class bachelor degree to integrate a master program. And it is the same for jobs.	education as investment	HE as a tool to success	Career success as individual mobilty

And its mainly true as the people who engage more with the course and make the most out of their degree are the ones who will probably access the better opportunities and jobs later. From what I have experienced, the people who are more successful around me did get an education which allowed them to get better jobs, earn more money and thus live out the life they want, whereas those who weren't fortunate enough to be educated to a higher degree ended up in very low-paying more physically demanding jobs that don’t allow them to live a fulfilling life.	education as investment	HE as a tool to success	Career success as individual mobilty
Because educated people are more skilled, versatile, resilient, informed, better prepared to pursuit their goals (…)	education as investment	HE as a tool to success	Career success as individual mobilty
I have a provable understanding of my chosen subjects – and being able to aide children with language acquisition is a big advantage. But If I pursue a corporate career, its also a great stepping stone for work in publishing or public relations. In todays society where degrees are a standard, it definitely helps. Good education often comes with valuable skills that employers look for. But considering the definition of success is different for everyone, I think education is only the stepping stone to success.	education as investment	HE as a tool to success	Career success as individual mobilty

I would like to think so because from my time studying at university my knowledge has grown and with a permanent qualification this should differentiate me from other candidates that apply for the same job.	education as investment	HE as a tool to success	Career success as individual mobilty
Yes, I definitely don't think I'd be able to get into the careers I want without this degree. I think university is a good stepping stone to getting where you want to be, but every university is different.	education as investment	HE as a tool to success	Career success as individual mobilty
I need university to get where I want to go so success here is really important for me to get where I want to go.	education as investment	HE as a tool to success	Career success as individual mobilty
From my life experiences I do think education is key to success as I have always worked hard and always been rewarded from this (...) I had a huge range of opportunities going to university whereas a lot of my friends didn't have much choice regarding course and location since they were limited with bad grades	education as investment	HE as a tool to success	Career success as individual mobilty
That is actually quite a difficult question. For myself, I would say absolutely, yes. Education will give me everything I essentially want in life: a great career in a job I enjoy, independence. That, for me, is success in life, because it will make me happy.	education as investment	HE as a tool to success	Career success as individual mobilty

As I am just an undergraduate it is difficult for me to give a answer to this but it is my opinion that having the educational experience does give you a good chance at succeeding.	education as investment	HE as a tool to success	Career success as individual mobilty
I think education is the foundation on which we build a successful life. Education itself must be used to attain success.	education as investment	HE as a tool to success	Career success as individual mobilty
Yes as if it was not for my course I would not be able to do what I want and for the specific programme you have to have a university degree so definitely my degree is very valuable towards my career life that will follow.	education as investment	HE as a tool to success	Career success as individual mobilty
If I want to do further studies, entering university is a must, so yes. It is necessary to learn as much as possible so as to contribute to the current forefront of research. Even for entering the workforce, jobs I am looking at require university education and a base level of math that can only be attained in university. And, especially in recent times, having a university degree is becoming a norm. My parents do not have degrees, but in their time, this was normal, and so success was achievable without one. The types of jobs today increasingly require a degree as a prerequisite	education as investment	HE as a tool to success	Career success as individual mobilty

I belive it will help me to get a good life and secure a job in wa world where getting a job without a higher or further education is hard (...) Yes , belive that what I achive at uni will help me to do well in my career ad what learn here can be apllied in my future career.	education as investment	HE as a tool to success	Career success as individual mobilty
Internships in corporations are harder to come by than ever, so Id be happy to continue in my childcare career – either by getting more education in this field, or just continuing my aupair experience (…)	perceptions of unemployment	Career success and perceptions of unemployment: Social and contextual constraints	Career success as individual mobilty
